# *In Vitro* and *In Silico* Evaluation of the Trypanocidal Activity of a Subfraction Isolated from *Mutisia campanulata*

**DOI:** 10.1021/acsomega.5c08989

**Published:** 2026-01-23

**Authors:** Grazielle Pereira da Silva, Lucas Resende Dutra Sousa, Paula Melo de Abreu Vieira, Ricardo Stefani, Andréa Mendes do Nascimento

**Affiliations:** † Department of Chemistry, Federal University of Ouro Preto (UFOP), Ouro Preto 35400-000, MG, Brazil; ‡ Phytotechnology Laboratory, School of Pharmacy, Federal University of Ouro Preto (UFOP), Ouro Preto 35400-000, MG, Brazil; § Morphopathology Laboratory, Department of Biological Sciences, Federal University of Ouro Preto (UFOP), Ouro Preto 35400-000, MG, Brazil

## Abstract

In the present study, a subfraction rich in aliphatic hydrocarbons and pentacyclic triterpenes was isolated after chromatographic steps from *Mutisia campanulata* Less and its trypanocidal activity were evaluated using *in vitro* and *in silico* approaches. The chemical structures of the compounds present in the subfraction were defined by GC–MS and ^13^C NMR data analysis. The trypanocidal activity *in vitro* of the subfraction indicated potency against epimastigote forms of the Y strain from *Trypanosoma cruzi* (IC_50_ of 5.88 ± 0.13 μg/mL). Molecular docking studies were conducted using four *T. cruzi* enzyme targets (1TC1, 1YHL, 2EF6 and 4C27) and six compounds, including a control. The pseudotaraxasterol obtained better results with an energy of −10.2 kcal/mol for the 4C27 enzyme. The RMSD trajectory of the pseudotaraxasterol in protein–ligand complex indicates stability during the 100 ns molecular dynamics simulation and several weak van der Waals interactions predominate in protein–ligand interactions. MMPBSA-per-residue decomposition analysis was used to provide insight into the interactions between the binding site and the pseudotaraxasterol. The findings revealed that the amino acid residues MET-106, LEU-356, and TYR-103 play a critical role in effective binding interactions. Therefore, pseudotaraxasterol holds potential for the development of new prototypes for the treatment of Chagas disease.

## Graphical abstract

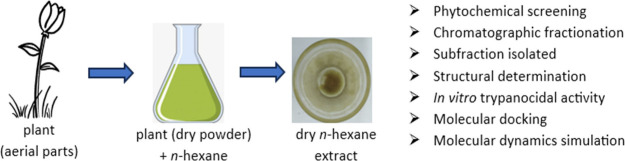

## 1 Introduction

Chagas disease affects over seven million people especially in developing countries, and its etiologic agent is the *Trypanosoma cruzi*.^1^ The hematophagous insect of the Reduviidae family in the Hemiptera order (subfamily triatominae) is the vector of this disease, which is endemic throughout Latin America, particularly in rural regions. The main source of infection in these nations is the parasite-contaminated feces of the vector. Other options include organ transplantation, blood transfusion, congenital transmission, and accidental consumption.^2^ This truth, coupled with the constant migration of individuals from endemic areas, means that Chagas disease has also been a concern in nonendemic places such as Europe, the United States, Canada, Japan and Australia, in recent decades.^3^ The disease’s clinical progression starts with an acute phase, which is marked by high parasitemia and detectable parasites in the blood, precedes the asymptomatic or symptomatic chronic phase, which has varying degrees of severity and progression.^4^ The acute phase of the infection is usually asymptomatic, and about 5% of patients have modest symptoms, such as fever, malaise, and the typical unilateral edema of the eyelids that happens when the insect bites close to the eye, commonly known as the Romana sign.^5^ In the chronic phase, approximately 30% of infected individuals develop cardiac problems such as cardiomegaly, heart failure, and arrhythmias are frequent, whereas megacolon or megaesophagus may result from gastrointestinal involvement.^6^

*T. cruzi* is a target organism of extreme importance in the development of new drugs. The only drugs widely used to treat the disease are nifurtimox and benznidazole, which are very effective, when given at the early stages of the infection.^7^ These drugs have some disadvantages, including little effect modifying the disease in the chronic phase, contraindicated during pregnancy, adverse effects during their use, and the resistance that the parasite has developed to their activity.^8,9^ There is, consequently, an urgent need for novel alternatives and effective treatments for this disease.^10^ In this context of therapeutic shortages, the search for new compounds from natural sources, such as plants, becomes a rational and promising strategy. The Asteraceae family, one of the most common groups of plants used in folk medicine worldwide, emerges as an interesting source for new trypanocidal compounds.^11^ Plants from the Asteraceae have yielded leading compounds against *T. cruzi*. These compounds are primarily terpenoids (especially sesquiterpene lactones) and flavonoids.^12,13^

The genus *Mutisia* (family Asteraceae, tribe Mutisieae) comprises 63 endemic species to South America, which are located in two well-defined areas: along the Andes, from northern Colombia to southern Chile and Argentina (Patagonia), and 11 species inhabit southern Brazil and adjacent regions of Paraguay, Uruguay, and northeastern Argentina.^14–16^ Several of these species are used in South American folk medicine for the treatment of cancer, gastric ulcers, respiratory diseases, heart diseases, hysteria, epilepsy, against chronic coughs and stomach pains.^17^ *Mutisia campanulata* Less is a liana popularly known as “winged divine carnation”, and occurs in Argentina, Paraguay and Brazil, in the states of Rio Grande do Sul, Santa Catarina, Paraná, Minas Gerais, Rio de Janeiro and São Paulo.^18^ To our knowledge, there is only one previous report on *M. campanulata* regarding the moderate antiproliferative activity of the *n*-hexane extract to glioblastoma cell lines, SF-295, with 58.3% of cell proliferation inhibition, and this study is from our research group.^19^ In an attempt to identify the compounds present in the *n*-hexane extract responsible for the antiproliferative activity, column chromatography was performed. A subfraction showed white crystals and was subjected to analysis by GC–MS and by ^13^C NMR and the result indicated that it was a mixture of aliphatic hydrocarbons and pentacyclic triterpenes. This significant class of secondary metabolites has been an important source of new substances to combat human parasitology^20,21^ and then, an *in vitro* screening for trypanocidal activity of the subfraction was considered. Pentacyclic triterpenes are widely distributed in the plant kingdom and have been largely isolated as isomeric mixtures.^22^ The aim of the present study was to investigate the trypanocidal activity of the isolated subfraction from *n*-hexane extract of the aerial parts of *M. campanulata*, rich in aliphatic hydrocarbons and pentacyclic triterpenes. Computational methods are frequently employed to lower the expenses related to developing novel drugs that have the capacity to bind particular molecular targets that are crucial to *T. cruzi* metabolism. Numerous researchers have examined the inhibition of proteins or enzymes that have been previously confirmed to be viable targets for *T. cruzi* and support this promising approach.^23^ In this scenario, the objective of the present study also was to evaluate the trypanocidal activity *in silico* potential of the pentacyclic triterpenes identified in the subfraction. The CYP51 (4C27) target showed the lowest average binding energy (−9.0 kcal/mol), indicating it is the most susceptible target among those tested. Information regarding the validation of the protein structure (PDB ID: 4C27) has been added, including resolution details, identification of missing residues, confirmation of active sites, and validation metrics such as Ramachandran plot statistics. Pseudotaraxasterol achieved the best individual result, with a binding energy of −10.2 kcal/mol for the CYP51 (4C27) target, surpassing the control (2-benznidazole, which ranged from −5.0 to −7.0 kcal/mol). Docking results indicated that the natural compounds, especially pseudotaraxasterol, demonstrated stronger affinities (more negative energy values) with at least one enzyme compared to 2-benznidazole. Pseudotaraxasterol emerged as the most promising candidate for detailed *in silico* investigations. The 100 ns MD simulation for the CYP51 pseudotaraxasterol complex showed a stable root-mean-square deviation (RMSD) (variation ΔRMSD ± 0.1 nm), indicating that the binding is favorable and remained stable throughout the simulation. The control (2-benznidazole) demonstrated inferior performance and greater instability (RMSD oscillating between 0.4 and 0.8 nm, and up to 1.0 nm at 310 K) compared to pseudotaraxasterol, making it a less promising ligand in terms of interaction stability. Principal Component Analysis (PCA) suggested an induced-fit mechanism at 310 K (physiological temperature), where the complex converges to a unique and well-defined conformational state, which is a strange trait for a potent inhibitor. Energy analyses and MD snapshots revealed that the interactions between pseudotaraxasterol and the CYP51 (4C27) protein are predominantly hydrophobic (van der Waals forces). The calculation of the average total binding free energy was −157.8 kJ/mol. van der Waals forces were the main favorable contributor (−200.5 kJ/mol). Polar solvation had a significant unfavorable contribution (+67.2 kJ/mol), typical for hydrophobic ligands. Residue decomposition analysis confirmed the significance of nonpolar interactions, highlighting residues MET-106, LEU-356, and TYR-103 as key contributors to binding stabilization. Pseudotaraxasterol was optimized using Density Functional Theory (DFT) calculations to refine its molecular geometry, analyze its electronic properties, and evaluate its frontier molecular orbitals (HOMO–LUMO).

## 2 Experimental Section

### 2.1 Plant Material, Extraction and Isolation

The aerial parts of *M. campanulata* were collected in Saramenha neighborhood, Ouro Preto city, State of Minas Gerais, Brazil, at flowering season, on October 25, 2011. A voucher specimen was deposited in the José Badini Herbarium of the Federal University of Ouro Preto (No. OUPR 26754). The registries in SisGen (Sistema Nacional de Gestão do Patrimônio Genético e do Conhecimento Tradicional Associado – National System of Management of Genetic Heritage and Associated Traditional Knowledge) were performed (A5133AE) according to the Brazilian legislation on access to biodiversity (Federal Law No. 13,123/2015). The aerial parts of *M. campanulata* were dried in a greenhouse with air circulation (40 °C) and powdered. The dry powder (41.5 g) was macerated at room temperature (23 °C) with *n*-hexane (Synth; 200 mL, 3 consecutive extractions over 24 h) to give the *n*-hexane (0.92 g) crude extract. The *n*-hexane extract (0.92 g) was subjected to column chromatography (CC) (2.3 × 50.0 cm; silica gel 60 0.063–0.200 mm, 70–230 mesh, Macherey-Nagel; ratio between sample and adsorbent 1:30) eluted with *n*-hexane–ethyl acetate 95:5, *n*-hexane–ethyl acetate 90:10, *n*-hexane–ethyl acetate 70:30, ethyl acetate, and ethyl acetate–ethanol 50:50 to give 40 fractions (ca. 12 mL each), which were monitored by thin layer chromatography (TLC) (silica gel GF_254_; Merck) and pooled into 15 subfractions (MCFr1-MCFr15). The subfraction MCFr5 (0.0720 g; white crystals) was subjected to analysis by GC–MS and by ^13^C NMR. Obtaining a crystalline solid often represents the successful isolation of a specific secondary metabolite from the complex mixture of the original plant extract. These analyzes indicated that the main compounds of the subfraction MCFr5 are aliphatic hydrocarbons and pentacyclic triterpenes. Since the MCFr5 subfraction is a mixture of compounds and has a low mass, subsequent separation of the compounds by chromatography suggests that the isolated compounds may have a low yield, making subsequent analysis by ^13^C NMR difficult. ^13^C NMR analysis would be difficult because there would not be enough sample material to generate a strong enough signal for detection and detailed structural determination.

### 2.2 Phytochemical Screening of Crude Extract

Qualitative phytochemical screening was carried on the *n*-hexane extract to identify their phytoconstituents, i.e., terpenoids, saponins, phenols/tannins, flavonoids and anthraquinones, using standard procedures.^24,25^ The tests were based on the color changes after the reaction of the extract with standard reagents to detect secondary metabolites.

### 2.3 Characterization of the Compounds Present in the MCFr5 Subfraction by GC–MS

The composition of the MCFr5 subfraction was determined by gas chromatography interfaced to a mass spectrometer (GC–MS) using a Shimadzu QP-2010 equipment. The following equipment and conditions were used: Phenomenex Zebron ZB-5MS column (30 m x 0.25 mm x 0.25 μm); helium (99.999%) carrier gas at a constant flow of 1.1 mL/min; 1 μL injection volume; injector split ratio 1:40; injector temperature 240 °C; electron impact mode at 70 eV; ion-source temperature 280 °C. The oven temperature was programmed from 100 °C (isothermal for 5 min), with an increase of 10 °C/min, to 250 °C (isothermal for 5 min), and 10 °C/min to 280 °C (isothermal for 15 min). The relative retention indices (RRI) for the compounds were determined according to the following equation and using *n*-alkanes as standards.^26^ RRI = 100n + 100­(tx – tn)/(tn + 1 – tn); where tx is the retention time of the constituent x; tn is the retention time of the *n*-alkane eluate before of the constituent x; tn + 1 is the retention time of the *n*-alkane eluded after of the constituent x and n is the number of carbon atoms in the *n*-alkane eluate before of the constituent x (see Supporting Information Figures S3 and S4). The RRI data was compared with those from pertinent literature.^27^

### 2.4 Characterization of the Compounds Present in the MCFr5 Subfraction by ^13^C NMR

The constituents of the MCFr5 subfraction were also determined by ^13^C NMR (see Supporting Information Figures S1 and S2). One-dimensional ^13^C NMR experiment was performed on a Bruker Avance III 400 MHz spectrometer (9.4 T). The experiment was performed at 100 MHz for ^13^C. Deuterated chloroform (CDCl_3_) was used as solvent and as an internal standard for calibrating the ^13^C NMR chemical shifts.

### 2.5 Trypanocidal Activity

Trypanocidal activity of the MCFr5 subfraction was measured using the previously described methodology.^28^ In general, epimastigotes of the *T. cruzi* Y strain were obtained during the exponential growth phase. The epimastigotes were then washed twice with LIT medium in sterile PBS (pH 7.2) and centrifuged at 1500 rpm for 10 min at 4 °C. Concentration was determined using a Neubauer chamber, and the epimastigotes were adjusted to a concentration of 5 × 10^6^/mL in LIT medium supplemented with 10% inactivated fetal bovine serum at 56 °C. To perform a screening of the mixture of pentacyclic triterpenes with possible biological activity against *T. cruzi*, the sample was incubated in 48 well plates containing 800 μL the suspension of parasites and 200 μL of the tested mixture (MCFr5 subfraction ) to obtain different final concentrations (50.0, 25.0, 12.5, 6.3, 3.2, 1.6, 0.8, and 0.4 μg/mL) diluted in sterile DMSO for 72 h. The parasites incubated in the absence of test mixture and in the presence of 1% DMSO were used as negative control and 2-benznidazole (50.0, 25.0, 12.5, 6.3, 3.2, 1.6, 0.8, and 0.4 μg/mL) as positive control. The activity was determined by counting in a Neubauer chamber and subsequent statistical analysis. The results were expressed as IC_50_ (concentration needed to decrease 50% in the number of parasites) and the tests were performed in triplicate. The IC_50_ values were obtained by linear regression analysis.

### 2.6 Molecular Docking

To better understand the trypanocidal activity, the identified pentacyclic triterpenes and 2-benznidazole (control) were subjected to molecular docking as ligands against *T. cruzi* molecular targets. The 3D chemical structures for each triterpene and control were prepared using Avogadro 1.95 software. Prior to docking, to refine the ligand geometries and analyze their electronic properties, Density Functional Theory (DFT) calculations were performed. The structures were optimized using the GAMESS software package. All calculations employed the B3LYP hybrid functional with the 6-31G­(d,p) basis set in the gas phase. Frequency calculations were performed at the same level of theory to confirm all optimized structures as true local minima. The energies of the Highest Occupied Molecular Orbital (HOMO) and Lowest Unoccupied Molecular Orbital (LUMO) were determined to evaluate the electronic properties of the compounds. These optimized structures and their corresponding partial charges were then used for the subsequent docking analyses. Thus, all simulations were performed using energy-minimized conformations. The molecular targets were prepared using AutoDockTools version 1.5.7, which involved the following standard procedures: adding polar hydrogens, removing artifacts from crystallography, and calculating Gasteiger–Huckel partial charges. Docking studies employed AutoDockVina^29^ software, which implements the Lamarckian genetic algorithm^30^ for the recognition of the appropriate binding sites and proper orientation of the ligand. For docking, the search grid was defined as 30 × 30 × 30 points centered on the crystallographic ligand of each target, thus ensuring that the binding site corresponded to the experimentally validated active site. The following crystallographically resolved *T. cruzi* molecular targets were selected: hypoxanthine phosphoribosyltransferase (HPRT, PDB ID: 1TC1, resolution 1.41 Å), farnesyl diphosphate synthase (FPPS, PDB ID: 1YHL, resolution 1.95 Å), dihydroorotate dehydrogenase (DHODH, PDB ID: 2EF6, resolution 2.10 Å), and cytochrome P450 CYP51 (PDB ID: 4C27, resolution 1.95 Å). The binding pocket (active site) was defined based on the position of the cocrystallized ligand [(*R*)-*N*-(3-(1*H*-indol-3-yl)-1-oxo-1-(pyridin-4-ylamino)­propan-2-yl)-2-fluoro-4-(4-(4-(trifluoromethyl)­phenyl)­piperazin-1-yl)­benzamide] found in the original PDB structure (PDB ID: 4C27). To validate the accuracy of our docking protocol, a redocking procedure was performed. The native cocrystallized ligand was first extracted from the complex and then redocked into the protein’s active site. The protocol was successfully validated, as the top-ranked pose reproduced the original crystallographic pose with a Root Mean Square Deviation (RMSD) of 1.09 Å (a value under the 2.0 Å threshold), confirming the protocol’s ability to identify the correct binding mode. Protein–Ligand Interaction Profiler software (PLIP)^31^ was employed to analyze the intermolecular interactions formed between the molecular targets and ligands. The construction of 3D diagrams was carried out using PyMOL. For cytochrome P450 CYP51 (PDB ID: 4C27), which showed to be the most promising target, more detailed validation was done. The stereochemical quality and geometric integrity of the protein structure were rigorously validated using the PROCHECK program, accessed via the SAVES (Structural Analysis and Validation Server) v6.0. This analysis was performed on the initial structure and again on the final structure obtained after 100 ns of molecular dynamics simulation to ensure the protocol did not compromise the protein’s quality and to ensure the reliability of the starting structure for docking analysis, its stereochemical quality was validated. The validation assessed the backbone dihedral angles (Φ and Ψ) by generating a Ramachandran plot, evaluating the percentage of residues in favored, allowed, and disallowed regions. The Ramachandran plot analysis (see Supporting Information Figures S5 and S6), performed with PROCHECK, confirmed the high quality of the initial model. The analysis showed that 92.9% of residues were in the ‘most favored’ regions, 6.9% in ‘additional allowed’ regions, and only 0.1% (1 residue) in the ‘disallowed’ region. This validation was repeated on the final structure after the 100 ns MD simulation to confirm its stability. The final structure maintained its high quality, with 93.1% of residues in the ‘most favored’ regions, 6.6% in ‘additional allowed’ regions, and only 0.3% (2 residues) in the ‘disallowed’ region. In both cases, the percentage of residues in the most favored regions is well above the 90% threshold typically expected for a high-quality, high-resolution model. This demonstrates that the protein structure is stereochemically valid and remained stable throughout the simulation process, validating its use for subsequent interaction analyses.

Furthermore, the protein crystal structure (PDB ID: 4C27) was initially inspected to identify residues lacking atomic coordinates. Analysis of the PDB file header, specifically the REMARK 465 section, revealed multiple structural gaps. These gaps were primarily located in highly flexible regions, such as loops and the N- and C-termini. As structural integrity is mandatory for subsequent docking and molecular dynamics studies, a complete protein model was generated. The coordinates for the missing residues (A:21–28, B:21–29, A:252–257, B:252–257, and A:480–487, B:480–487) were built and refined using the Prastool software.^32^

### 2.7 Molecular Dynamics Simulation

Pseudotaraxasterol, which showed the greatest binding affinity (−10.2 kcal/mol to 4C27) was submitted to molecular dynamics simulation using GROMACS 2024.2 molecular dynamics software^33^ and CHARMM36 force field. The simulation aimed to understand the effects of time, conformation, energy, and temperature on the binding affinity of the ligand to the molecular target. Therefore, 4C27 was downloaded from the RCSB (https://www.rcsb.org/). As the downloaded structure had missing heavy and hydrogen atoms, it was repaired with the PRAS Tool;^32^ then, it was solvated in the TIP3 water model using the CHARMm force field (https://www.charmm.org/) with an explicit spherical boundary with a harmonic restraint model. A counterion (NaCl) was added to the system to neutralize the surface charge of the molecular target. The ligand was also prepared using the CHARMm force field via the CGenFF Web site (https://cgenff.com) and further combined with the molecular target to finally form the complex to be simulated. Then, the system was heated to the target temperature (298 and 310 K) for 1 ns with 2 fs time step to pull back the system from the local minima. The nonbonded interaction cutoff was set to 10 Å, and the spherical cutoff method was used to treat long-range electrostatic interactions. Subsequently, the system was equilibrated for 10 ns at the target temperature with the previously described parameters and a C-rescale barostate prior to the production phase. Periodic boundary conditions were applied in all directions, and all bonds were constrained using the LINCS algorithm. The production time was 100 ns and was conducted on an *NPT* ensemble, keeping the pressure and temperature constant at 1.0 bar using the Parrinello–Rahman and V-rescale algorithms, respectively. The simulation was performed on a system configured with an Intel Core i5-11400H CPU, 16 GB RAM, a NVIDIA RTX 3060 GPU, and a Linux system. Finally, the trajectory file was analyzed for changes in the root-mean-square deviation (RMSD) and hydrogen bond count. The g_mmpbsa tool,^35^ which implements the MMPBSA method^36^ for GROMACS, was used to calculate the binding energy of the protein–ligand complex.

To analyze the dominant conformational motions at functionally relevant temperatures, Principal Component Analysis (PCA) was performed on the MD simulations. The analyses were conducted at 298 K (25 °C), to correlate with *in vitro* assay conditions, and at 310 K (37 °C), to simulate the *in vivo* physiological environment of human infection. The PCA was executed using the MDAnalysis, scikit-learn, and matplotlib Python libraries. Structural data for each simulation frame was extracted from GROMACS trajectory files (.xtc) and associated topology files (.pdb) using MDAnalysis package.^37^ Atom positions for all frames were gathered and reshaped into a two-dimensional array, where rows correspond to time frames and columns to atomic coordinates. Prior to PCA, the atomic coordinates were normalized using z-score scaling with StandardScaler from scikit-learn. This step ensures that all features contribute equally to the analysis. Classical PCA was then applied using scikit-learn’s PCA class, which computes the covariance matrix and determines its eigenvectors and eigenvalues. The resulting principal components represent the dominant collective motions of the protein–ligand complex throughout the simulation. Data were projected onto the first two principal components to visualize the main conformational transitions. This approach allows for a robust, reproducible analysis of structural dynamics and conformational heterogeneity in response to temperature and the interaction of the protein–ligand (pseudotaraxasterol) complex.

## 3 Results and Discussion

### 3.1 Characterization of the Compounds Present in the MCFr5 Subfraction by GC–MS

The MCFr5 subfraction, originating from the chromatographic fractionation of *n*-hexane crude extract from *M. campanulata*, was subjected to analysis by GC–MS to obtain the volatile profile. The relative retention indices (RRI) were calculated by comparing the retention times of each compound and a series of *n*-alkanes (linear hydrocarbons) under identical gas chromatography conditions. Alkanes were used as reference points in the calculation of RRI. The identity of the compounds, present in the MCFr5 subfraction, was confirmed by comparison of their RRI with literature values. This method standardizes retention times, making them less dependent on specific instrument conditions, which allows for more reliable compound identification by comparison to known data.

The results, described in Table 1, showed nine compounds present in the MCFr5 subfraction, but only three aliphatic hydrocarbons were identified: nonacosane (2.22%), untriacontane (17.54%) and tritriacontane (1.04%). The three aliphatic hydrocarbons were identified by GC–MS because the calculated RRI was compared with RRI values established in scientific literature, as a match indicates the same compound under similar analytical conditions (Table 1). For the other compounds, the RRI was not found in scientific literature,^27^ not allowing identification.

**Table 1:** Phytochemical Constituents Identified in the MCFr5 Subfraction by GC–MS

Peak	RRI^a^	RRI^b^	Constituent	Area (%)
1	2900	2899	Nonacosane	2.22
2	3100	3101	Untriacontane	17.54
3	3300	3300	Tritriacontane	1.04
4	Not found	3334	Unidentified	27.43
5	Not found	3359	Unidentified	1.31
6	Not found	3382	Unidentified	27.24
7	Not found	3423	Unidentified	0.31
8	Not found	3471	Unidentified	9.61
9	Not found	3483	Unidentified	13.30

a Relative Retention Indices; see Adams.^27^

b Relative Retention Indices on ZB-5MS column (relative to *n*-alkanes).

### 3.2 Characterization of the Compounds Present in the MCFr5 Subfraction by ^13^C NMR

The MCFr5 subfraction also was subjected to analysis by ^13^C NMR to try to identify compounds not identified by GC–MS. The following pentacyclic triterpenes were identified: α-amyrin, β-amyrin, taraxasterol, pseudotaraxasterol and lupeol (Figure 1). The identification was carried out by comparison of experimental ^13^C NMR chemical shifts with literature data.^38–40^ For this reason, instead of presenting a detailed discussion of the results, only some peculiarities are presented. Structural determination of pentacyclic triterpenes uses ^13^C NMR to identify the basic skeleton and functional groups by comparing observed chemical shifts. Key signals include those for olefinic and carbinol carbon atoms. The ^13^C NMR spectrum shows signals attributed to olefinic carbon atoms of pentacyclic triterpenes with urs-12(13)-ene type skeleton (compound 1: δC 124.4 and 139.6 ppm, for C-12 and C-13), olean-12(13)-ene type skeleton (compound 2: δC 121.7 and 145.2 ppm, for C-12 and C-13), taraxast-20(30)-ene type skeleton (compound 3: δC 107.1 and 154.6 ppm, for C-30 and C-20), taraxast-20(21)-ene type skeleton (compound 4: δC 118.9 and 139.8 ppm, for C-21 and C-20) and lup-20(29)-ene type skeleton (compound 5: δC 109.3 and 150.9 ppm, for C-29 and C-20). The signal at δC 79.0 ppm was attributed to carbinol carbon atoms (for all triterpenes). In conclusion, we were able to obtain complete NMR spectral assignments for the five triterpenes 1–5, allowing the unambiguous assignment of the structures of the compounds.

**Figure 1:**
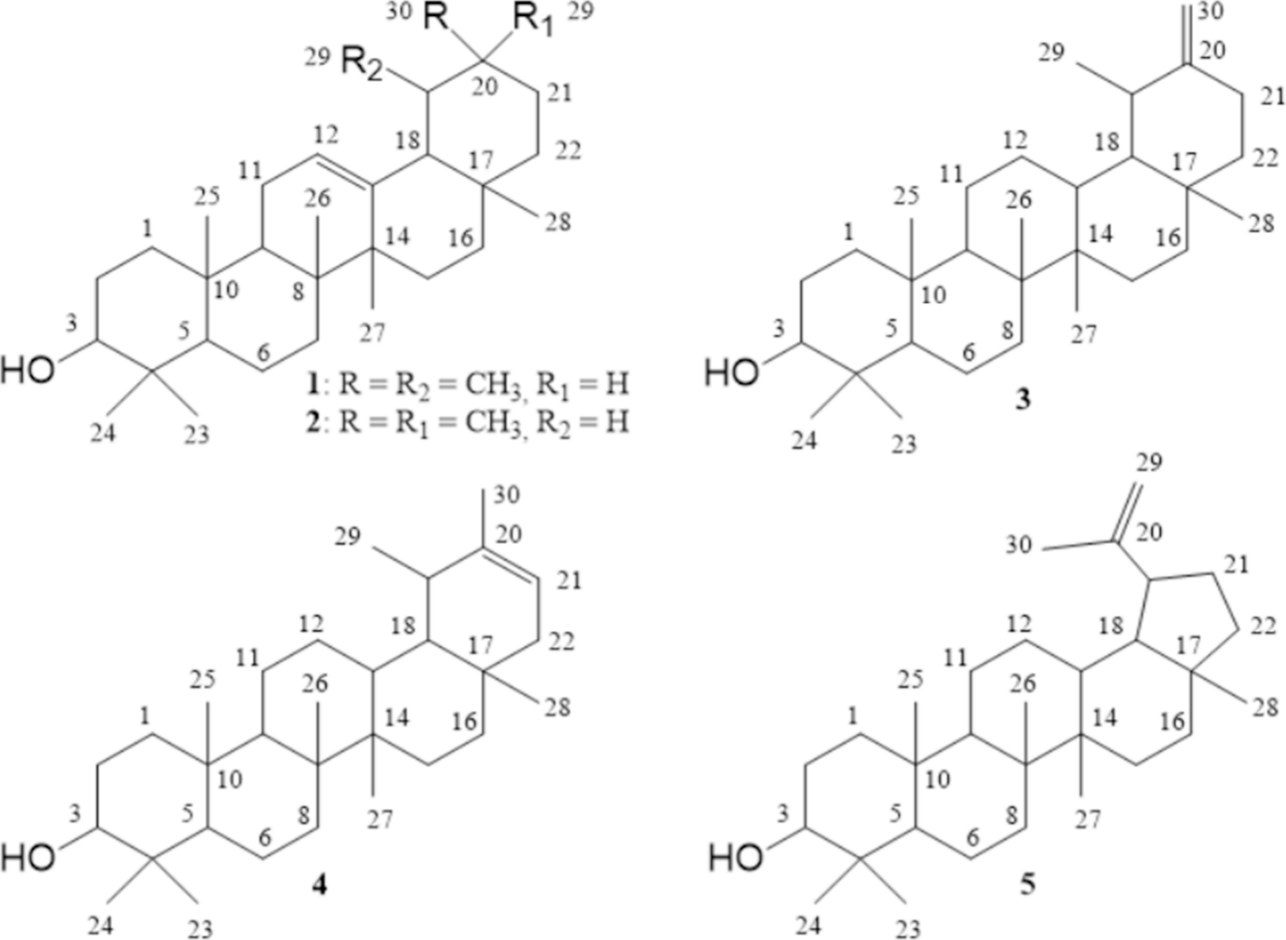
Structural representations for α-amyrin (1), β-amyrin (2), taraxasterol (3), pseudotaraxasterol (4) and lupeol (5) present in the MCFr5 subfraction isolated from *M. campanulata*.

The five pentacyclic triterpenes and the three aliphatic hydrocarbons are reported for the first time by the species *M. campanulata*. Howewer, they may not be used to differentiate *M. campanulata* from others in the genus, because β-amyrin, lupeol and pseudotaraxasterol have already been isolated from the *n*-hexane extract of the aerial parts of *M. acuminata*.^41^ There are many pentacyclic triterpenes identified in the genus *Mutisia* among which, those with a carboxyl group at the C-28 position and those oxygenated at C-3 position, have been reported.^42,43^ Previous phytochemical investigations on the genus *Mutisia* have reported the isolation of several kinds of compounds.

### 3.3 Phytochemical Screening of Crude Extract

Phytochemical screening of crude *n*-hexane extract reveals the presence of terpenoids and a subfraction rich in pentacyclic triterpenes was isolated from this extract, corroborating the results of the screening. Saponins, phenols/tannins, flavonoids and anthraquinones were not detected.

### 3.4 Trypanocidal Activity

*In vitro* evaluation screening of the MCFr5 subfraction trypanocidal effect was performed using the extracellular epimastigotes because of their simple culture, without the necessity of cell infection or coculture with other cell lineages. It is widely used to study the trypanocidal effect of new drugs, because of its morphological and biochemical similarities to trypomastigotes, and is also easily applied to the investigation of trypanocidal mechanisms of substances.^44^ The results of trypanocidal activity are described in Table 2. The lower the IC_50_ value, the better the trypanocidal activity, as it means that we would only need a minimum amount sample to smooth 50% of the parasites. The MCFr5 subfraction, which contains a mixture of pentacyclic triterpenes, presented trypanocidal activity with relatively low IC_50_ values (5.88 ± 0.13 μg/mL) however, the IC_50_ values are still higher than those of the positive control (2-benznidazole). Chagas disease is a devastating and neglected illness prevalent in 21 countries across Latin America. Pentacyclic triterpenes have been investigated as potential drug candidates for Chagas disease.^21^ For example, pentacyclic triterpenes were isolated from extracts of *Miconia* species and the ursolic, oleanolic and gypsogenic acids showed trypanocidal activity, with ursolic acid being the most active.^45^ In this study, they reached the conclusion that the presence of a free hydroxyl group at C-3 and/or the carboxyl group at C-17 is required for trypanocidal activity. Furthermore, the double bond between C-12 and C-13 may also play a role in the activity because the pentacyclic triterpene friedelanol lacking any double bond was inactive despite the presence of a hydroxyl group at C-3.^21^ In another research, pentacyclic triterpenes isolated from *Austroplenckia populnea* and four compounds of known anti *T. cruzi* activity were tested.^46^ Of those triterpenes tested 20-hydroxy-tingenone showed high activity, epikatonic acid was less active, while populnilic and populninic acids were inactive against the trypanosome of the subgenus *Schizotrypanum* tested. The tingenone (maitenine), that differs from 20-hydroxy-tingenone only by the presence of a methyl instead of the hydroxyl group at 20-position, inhibited *T. cruzi* growth mainly by DNA double-strand intercalation mechanism. The research inferred that the presence of a carboxylic group at the 20-position, such as in epikatonic acid, populnilic acid or populninic acid rises loss of activity, possible by difficulties in cross cell cytoplasmatic membrane. In this way, changes in critical radicals affect markedly the drug activity against *T. cruzi*. An *in vitro* screening was realized to examine the potential trypanocidal activity of 20 extracts obtained from 10 different plant species growing in the Brazilian Cerrado. This study examined the most active extracts (*n*-hexane extracts) and allowed the identification of α-amyrin, β-amyrin, lupeol and other triterpenes and sterols. The results showed that pure amyrins are inactive whereas the *n*-hexane leaf extract of *Tibouchina stenocarpa* was active.^47^ In addition, the triterpene pseudotaraxasterol has been reported to have weak *in vitro* trypanocidal activity with IC_50_ of 42.57 μg/mL against intracellular forms of *T. cruzi* (Tulahuen CL2, β-galactosidase, a drug-sensitive strain belonging to the discrete typing units, DTU VI).^48^ We were unable to find any record of taraxasterol trypanocidal activity in the literature. So, the trypanocidal effects of the mixture of pentacyclic triterpenes isolated in the present work may have been increased due to a synergistic interaction, rather than being ascribed by a specific compound.

**Table 2:** *In Vitro* Trypanocidal Activity of the MCFr5 Subfraction and 2-Benznidazol

Sample	IC_50_ (μg/mL)^a^
MCFr5 subfraction	5.9 ± 0.13
2-benznidazole (positive control)	1.2 ± 0.11

a Results are expressed as IC_50_: drug concentration to give 50% reduction in the number of epimastigotes of *T. cruzi*.

### 3.5 Molecular Dynamics and Docking

Molecular docking is typically utilized to assess the affinity and recognition of receptor–ligand complexes. Therefore, given the results for *in vitro* activity, molecular docking studies were conducted with the pentacyclic triterpenes identified in MCFr5 subfraction to obtain information on how these compounds are recognized by potential targets involved in trypanocidal activity. Hypoxanthine phosphoribosyltransferases are enzymes that are critical for the salvage of preformed purines and are essential for *T. cruzi*, as they allow the use of host purines for their survival, and blocking these enzymes may be a strategy for the development of new treatment methods for Chagas disease.^49^ The tripanossomal enzyme Farnesyl Diphosphate Synthase, which catalyzes the parasite’s manufacture of ubiquinones, heme, and sterols, is another one that is becoming a possible target for novel medications.^50^ Also essential for the survival of *T. cruzi*, the enzyme dihydroorotate dehydrogenase has emerged as a crucial molecular target for Chagas disease medication development.^51^ The cytochrome P450 CYP51 is an enzyme that is involved in ergosterol biosynthesis, which is essential for parasite membrane integrity. Inhibitors acting on CYP51 may improve antiparasitic efficacy and reduce the risk of resistance development.^52^ The knowledge of amino acid distribution at the active site of these *T. cruzi* molecular targets can be exploited to rationally design for their inhibitors.^50^

As shown in Table 3, the molecular targets examined included Hypoxanthine Phosphoribosyltransferase (1TC1), Farnesyl Diphosphate Synthase (1YHL), Dihydroorotate Dehydrogenase (2E6F), and cytochrome P450 CYP51 (4C27). The analysis was conducted using molecular docking simulations to determine the binding free energy between each compound and the target enzyme. A more negative energy value indicates a stronger affinity between the ligand and enzyme, suggesting a higher inhibitory potential. Six compounds were tested: a control (2-benznidazole), β-amyrin, taraxasterol, pseudotaraxasterol, lupeol, and α-amyrin. The control exhibited binding energies ranging from −5.0 to −7.0 kcal/mol. In contrast, all natural compounds showed stronger affinities (more negative values) with at least one enzyme, except for 1YHL, indicating better performance and potential inhibitory activity than the control.

**Table 3:** Docked Structure’s Binding Energies (kcal/mol) Found for 2-Benznidazole and Pentacyclic Triterpenes to Different *T. cruzi* Molecular Targets^a^

Ligand	1TC1	1YHL	2E6F	4C27
2-benznidazole	–6.1	–**6.8**	–5.0	–6.7
β-amyrin	–7.5	–5.8	–**6.6**	–9.7
taraxasterol	–8.0	–5.6	–6.5	–9.6
pseudotaraxasterol	–**8.3**	–5.4	–6.4	–**10.2**
lupeol	–7.7	–5.5	–6.3	–8.5
α-amyrin	–8.0	–5.4	–5.8	–9.3

a Bold text: a more negative energy value shows a stronger affinity between the ligand and the molecular targets.

The average binding energies obtained for each enzyme were −7.6 kcal/mol for 1TC1, −5.73 kcal/mol for 1YHL, −6.10 kcal/mol for 2E6F, and −9.0 kcal/mol for 4C27. Among these, the enzyme CYP51 (4C27) showed the lowest average energy values, indicating it is the most susceptible target among the ligands tested. The compound with the best individual result was pseudotaraxasterol, with an energy of −10.2 kcal/mol for the CYP51 (4C27) enzyme, closely followed by β-amyrin and taraxasterol, with −9.7 and −9.6 kcal/mol, respectively, for the same target. Figure 2 shows the binding on each target.

**Figure 2:**
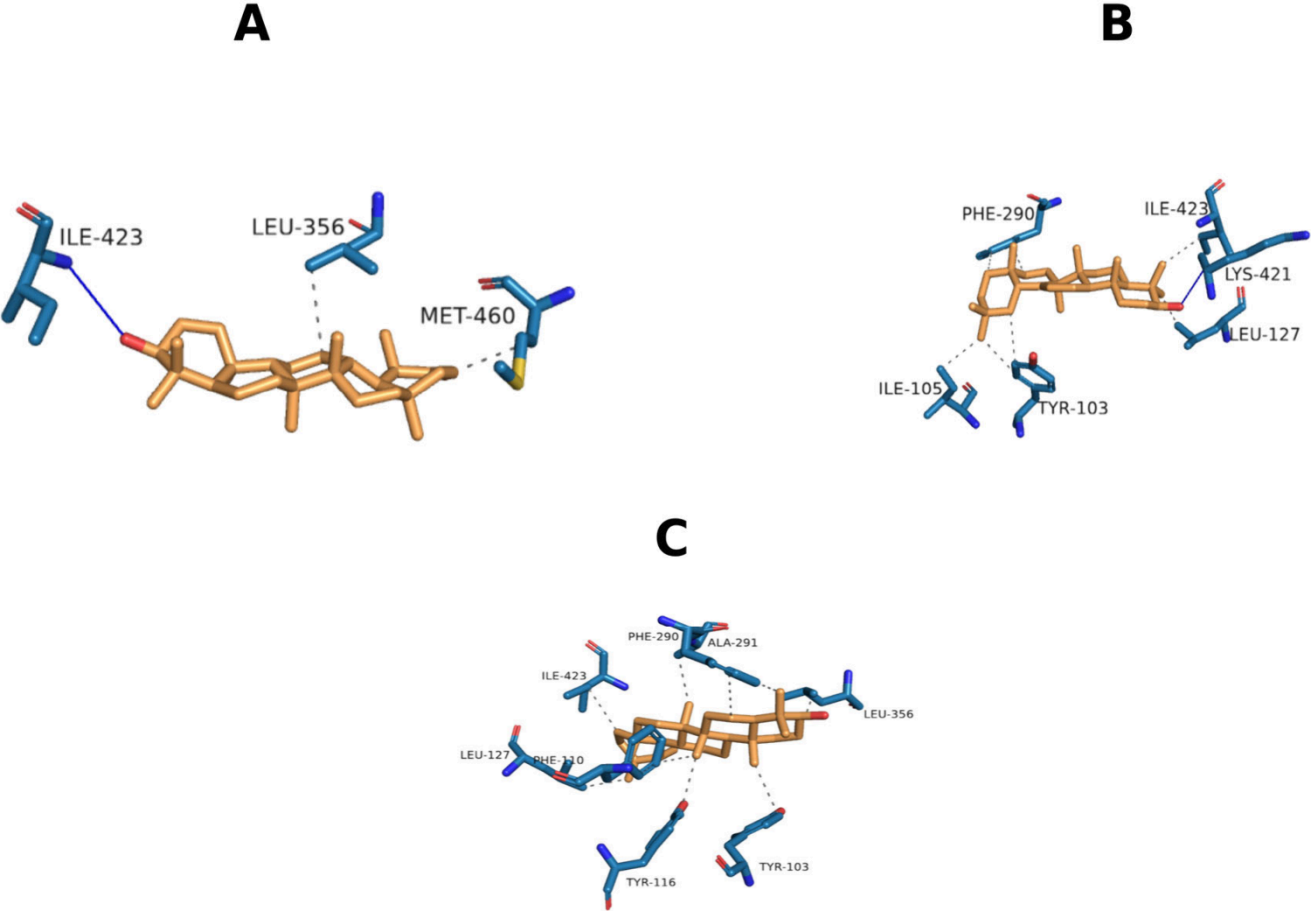
Representation of the intermolecular interactions found for 4C27 complexes with pseudotaraxasteol (A), β-amyrin (B) and taraxasterol (C). Carbons are represented in light blue (enzyme) and orange (ligand), nitrogen in dark blue and oxygens in red. Blue lines: hydrogen bond. Gray dashed line: hydrophobic interaction.

Figure 2 shows that the intermolecular interactions of the 4C27 complexes with β-amyrin (B) and taraxasterol (C) are only weak van der Waals forces, although β-amyrin also showed hydrogen bonding with the residue LIS-421 and the OH group at C-3. On the other hand, pseudotaraxasterol (A) exhibits a strong hydrogen bond with 4C27, involving residues ILE-423 and the OH group at C-3 and hydrophobic interactions with residues LEU-356 and MET-460. The data suggests that pseudotaraxasterol emerges as the most promising candidate among those tested, showing particular efficacy in binding to CYP51 (4C27), which is the most susceptible target for the ligands, indicating that it is the optimal choice for more detailed and comprehensive *in silico* investigations. Moreover, to better understand the electronic behavior of the ligands, DFT calculations were performed. The frontier molecular orbitals, HOMO and LUMO, are critical determinants of chemical behavior. The orbital distributions are shown in Figures S7 and S8. The energy gap is a key indicator of kinetic stability and chemical affinity. A small gap implies a molecule is more reactive and has a higher binding affinity, as it requires less energy to excite an electron. Our analysis revealed that 2-benzinadole possesses a significantly smaller energy gap (4.219 eV) compared to pseudotaraxasterol (7.469 eV). This suggests that 2-benzinadole is electronically more active and a better electron donor, potentially enhancing its ability to form key interactions (such as charge-transfer) within the protein’s active site via polar and π- π interactions. In contrast, the large gap of pseudotaraxasterol indicates high chemical stability and that it interacts with the protein via nonpolar and van der Waals interactions.

Therefore, molecular dynamics simulation studies were performed to determine the stability of the interaction between CYP51 (4C27) and pseudotaraxasterol. The trajectory results obtained from the 100 ns molecular dynamics simulation were analyzed for root-mean-square deviation (RMSD), hydrogen bonding, and energy terms. Initially, an RMSD examination was performed to evaluate the stability of the simulations. The RMSD plot (Figure 3) for pseudotaraxasterol in protein ligand complex was almost stable and had no significant variation during all 100 ns of simulation, the variation of the RMSD value during the course of simulation was found to be less than 0.3 nm (ΔRMSD is ± 0.1 nm) at both temperatures. Thus, the binding remained stable throughout the 100 ns molecular dynamics simulation run without any significant variance. This result suggests that the binding between pseudotaraxasterol and the ligand is favorable and was stable during the overall run.

**Figure 3:**
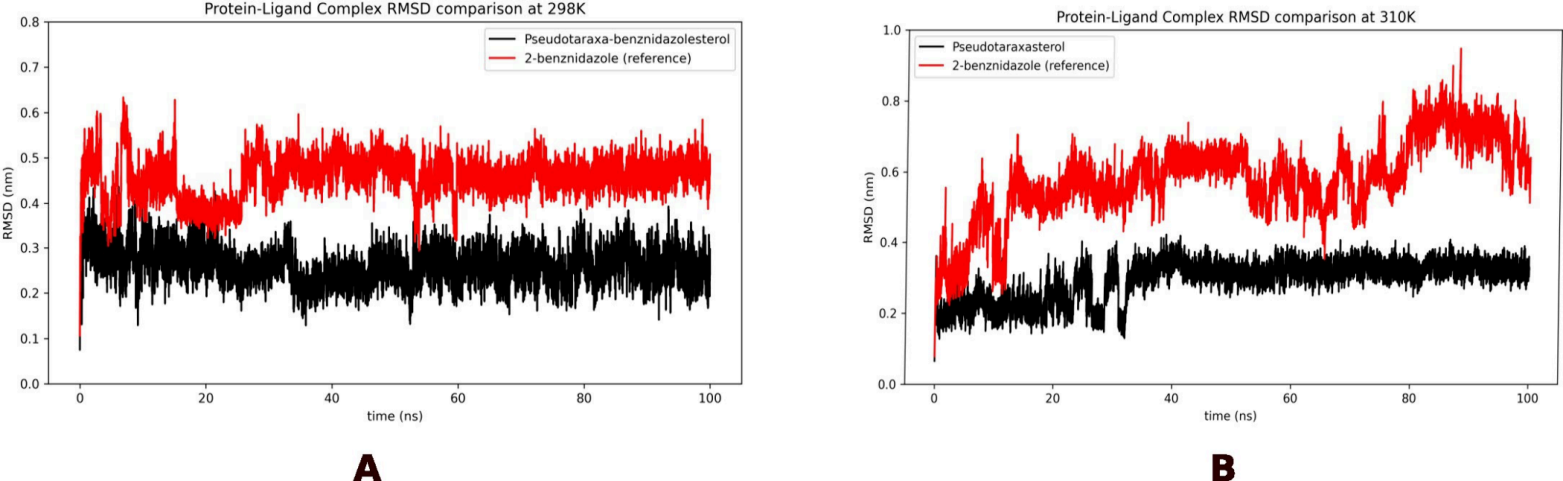
RMSD comparison of the protein bound to pseudotaraxasterol (black) and 2-benznidazole (reference, red) at 298 K (A) and 310 K (B).

The RMSD for 2-benznidazole, represented by the red line, demonstrated an inferior performance compared to pseudotaraxasterol. At 298 K, the complex with 2-benznidazole exhibited significantly higher RMSD values, oscillating between approximately 0.4 and 0.8 nm (ΔRMSD is ± 0.3 nm). This becomes more pronounced at 310 K. Under this condition, the instability of 2-benznidazole is even more evident, with its RMSD reaching peaks close to 1.0 nm and showing erratic fluctuations. Therefore, the data conclusively indicate that 2-benznidazole forms a considerably more unstable complex with the protein compared to pseudotaraxasterol, making it a less promising ligand in terms of interaction stability, especially under physiological temperature conditions.

Figure 4 represents snapshots of the simulated configurations of the complex at times 0, 10, 40, and 100 ns. As shown in Figure 4 and Table 4, at the start of the simulations (0 ns), the ligand forms hydrogen bonds with residues ILE-423 and LYS-421, as well as hydrophobic interactions with residues VAL-356, LEU-356, ALA-251, MET-460, and PRO-210. During the simulation, the protein–ligand interactions were mainly hydrophobic and the residues interacting with the ligand changed over time; however, the interactions with the residues LEU-356 and TYR-103 were the most persistent during the simulation. These results indicate that the ligand shows a preference for TYR (tyrosine) and LEU (leucine) residues during the simulation. Finally, at 100 ns, the OH group at the ligand C-3 makes a hydrogen bond with the residue PHE-290. Although some strong hydrogen bonds have been reported to be responsible for the antiparasitic activity of pseudotaraxasterol,^53^ simulation results show that ligand-protein interactions for pseudotaraxasterol are mostly dominated by a series of weak van der Waals interactions, which is confirmed by MMPBSA results (Table 5).

**Figure 4:**
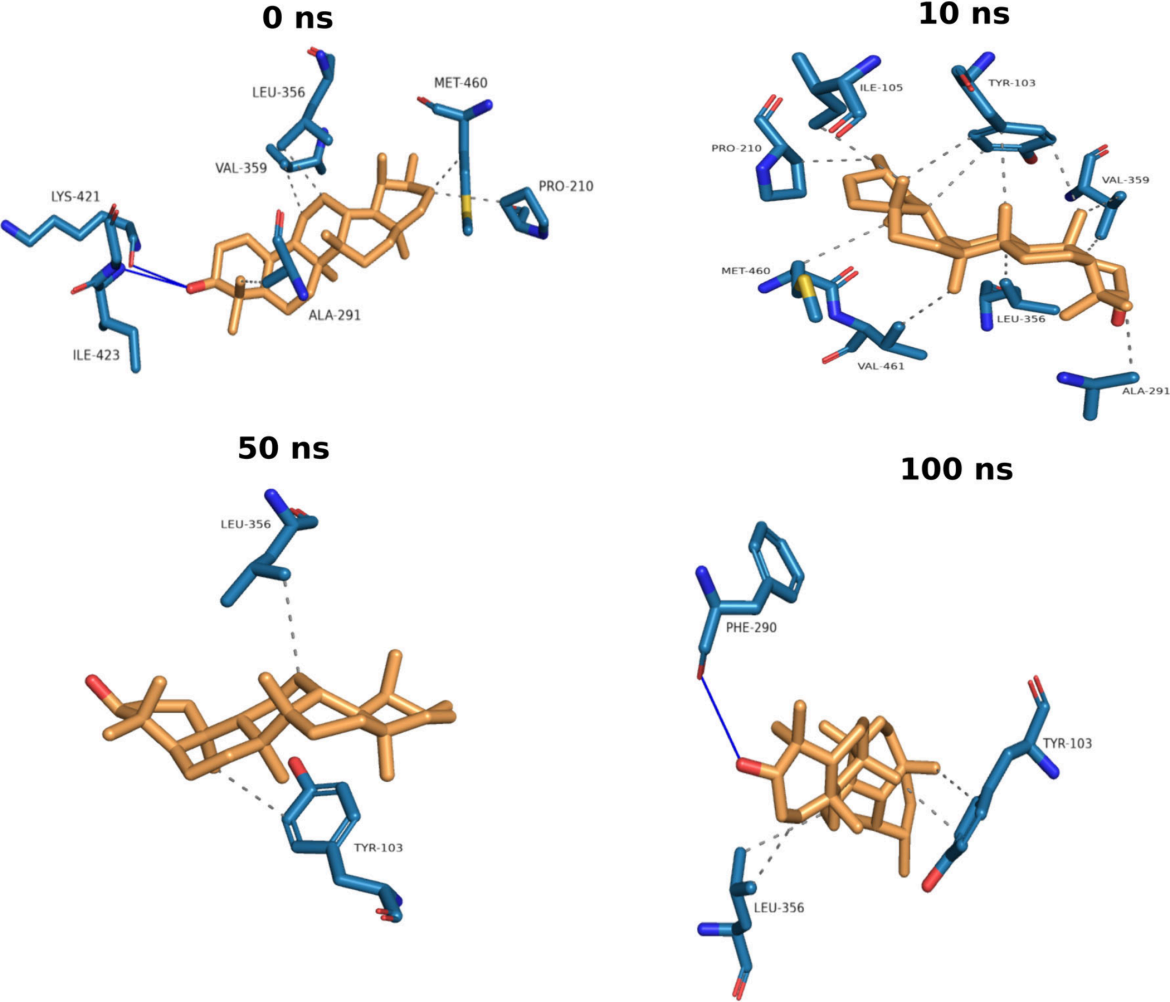
Snapshots representation RMSD of the pseudotaraxasterol-4C27 complex biding. The snapshots of ligand–protein complex at time = 0, 10, 50, and 100 ns. Carbons are represented in light blue (enzyme) and orange (ligand), and nitrogen in dark blue and oxygens are in red. Blue lines: hydrogen bond. Gray dashed line: hydrophobic interaction.

**Table 4:** Hydrophobic and Hydrogen Bond Interactions between Pseudotaraxasterol and the Molecular Target 4C27 ^a^

Time (ns)	Hydrophobic interactions	Hydrogen bond interactions
0	ALA-291, LEU-356, MET-460, PRO-210, VAL-359	ILE-423, LYS-421
10	ALA-291, ILE-105, LEU-356, MET-460, TYR-103, VAL-359, VAL-461	
50	TYR-103, LEU-356	
100	TYR-103, LEU-356	PHE-290

a Abbreviations: ALA, alanine; ILE, isoleucine; LEU, leucine; LYS, lysine; MET, methionine; PHE, phenylalanine; PRO, proline; TYR, tyrosine; VAL, valine.

**Table 5:** Summary of Binding Interaction Energy Components (kJ/mol) between Protein and Ligand, Showing Average ± Standard Deviation for van der Waals, Electrostatic, Polar Solvation, Nonpolar Solvation, and Total Energy

Energy components	Energy (kJ/mol)
van der Waals	–200.48 ± 10.98
Electrostatic	–2.21 ± 4.83
Polar solvation	+67.16 ± 10.43
Nonpolar solvation	–22.31 ± 10.43
Total	–157.85 ± 13.84

Furthermore, MMPBSA results show that the total binding free energy averaged −157.8 kJ/mol, with van der Waals (vDW) forces being the predominant favorable contributor (−200.5 kJ/mol), and nonpolar solvation also supporting binding (−22.3 kJ/mol). Electrostatic interaction energies were minor (−2.2 kJ/mol), indicating that specific charge–charge interactions play a limited role compared to hydrophobic and dispersive contacts in this complex. Polar solvation has a significant unfavorable contribution (+67.2 kJ/mol), showing the high energetic cost of desolvating polar groups upon binding, which is typical for hydrophobic ligands interacting with proteins.

Figure 5 shows the number of hydrogen bonds formed during the molecular dynamic simulation time. Hydrogen bond interactions are transitory, change over time, and play an important role in the stabilization of protein–ligand complexes. The hydrogen bonding profiles observed for the reference compound (2-benznidazole) and pseudotaraxasterol reveal distinct differences in their modes of molecular interaction throughout the simulation windows at both 298 and 310 K. The reference compound presents persistent and dynamic hydrogen bond formation, exhibiting fluctuations between 1 and 5 hydrogen bonds over time. These results indicate a significant contribution of polar interactions to the stabilization and flexibility of 2-benznidazole. At 310 K, the reference compound (2-benznidazole) exhibits reduction in hydrogen bond formation compared to its behavior at 298 K, suggesting lower interaction stability and increased molecular motion at the elevated temperature.

**Figure 5:**
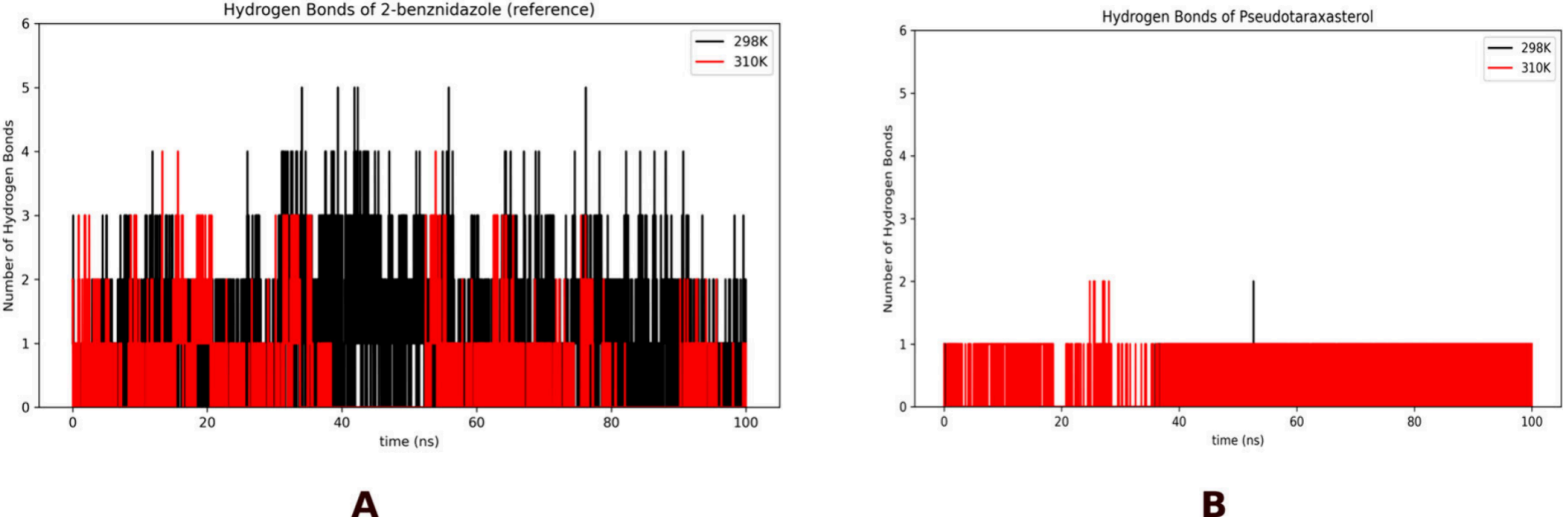
Time evolution of hydrogen bond formation during 100 ns molecular dynamics simulations for (A) 2-benznidazole (reference compound) and (B) pseudotaraxasterol at two temperatures, 298 K (black) and 310 K (red).

Pseudotaraxasterol demonstrates a reduced capacity for hydrogen bond formation at both temperatures, with only sparse and brief events observed at the start of the trajectory and almost no lasting contacts, thereafter, being reduced to only one stable hydrogen bonding until the end of simulation. Pseudotaraxasterol, as a pentacyclic triterpene, possesses a hydroxyl group at the C-3 position (3β-ol). This is consistent with previous literature noting that triterpene hydrogen bonding via C-3 is typically more effective when specific receptor or protein partners are present, whereas bulk interactions remain dominated by nonpolar interactions.^21^ The overall result of hydrogen bonds indicates that although there are hydrogen bonds that are important for the initial pseudotaraxasterol binding to 4C27, the interactions between the ligand and the protein are mostly dominated by van der Waals and other nonpolar interactions.

PCA analysis (Figure 6) can indicate the influence of temperature on the dynamics of the Pseudotaraxasterol-bound complex. At 298 K, the complex demonstrated high structural stability, as already indicated by the low and constant RMSD. However, the PCA at this temperature revealed the exploration of a relatively broad conformational space, suggesting that the complex interconverts between multiple, likely isoenergetic, conformational states. This behavior can be interpreted as a functional flexibility, where the ligand is bound stably, yet the complex as a whole retains a degree of dynamism. The MD at 310 K, a temperature mimicking physiological conditions, uncovered a more specific and optimized binding mechanism. The PCA at 310 K showed that, following an initial conformational rearrangement within the first ∼30 ns of the simulation, the complex converged into a single, well-defined conformational state, where it remained for the duration. This is quantitatively corroborated by the RMSD analysis, which displays a stable plateau with minimal fluctuations after the same transition period. Such behavior is characteristic of an induced-fit mechanism, wherein additional thermal energy allows the system to overcome minor energy barriers to reach a deep energy minimum, corresponding to a high-affinity and specific interaction. The resulting conformational rigidity at 310 K is a highly desirable trait for a potent inhibitor, as it implies a lower entropic penalty upon binding.

**Figure 6:**
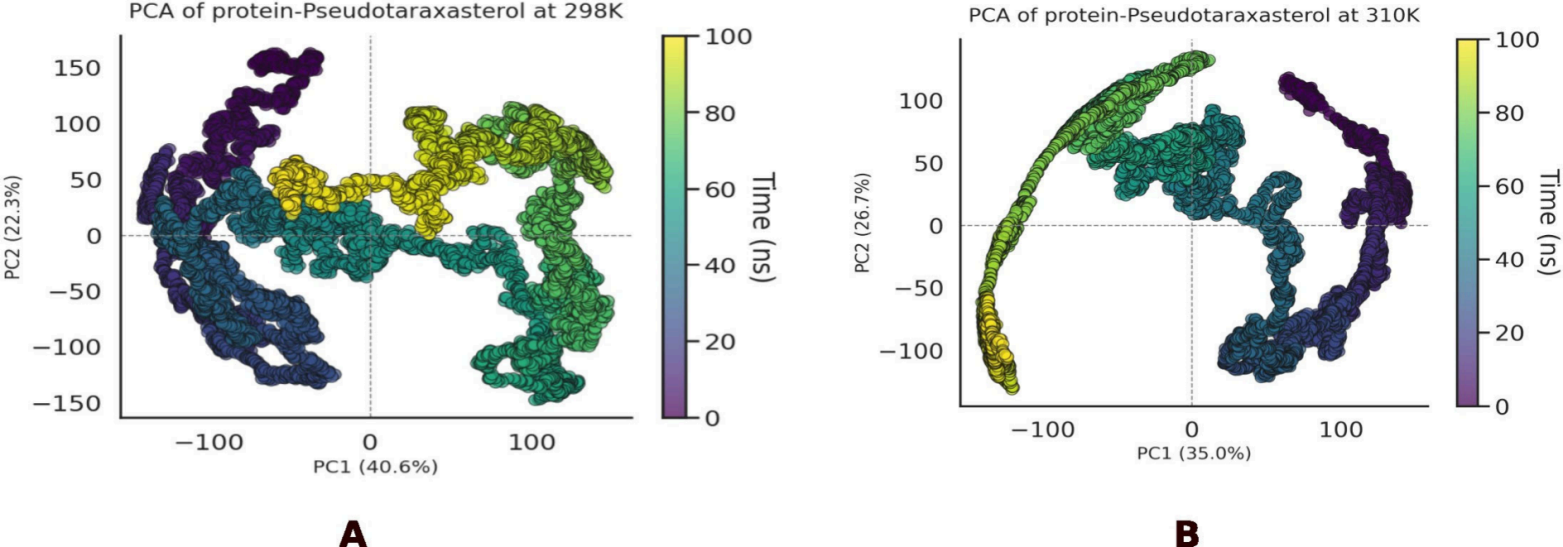
Principal Component Analysis (PCA) of the complex at 298 K (A) and 310 K (B). Each point represents a simulation frame, colored by time from 0 ns (purple) to 100 ns (yellow).

Moreover, to confirm the visual inspection of protein–ligand interactions (Figure 4), the residue decomposition analysis (Figure 7) was conducted through MMPBSA to investigate the key amino acids contributing to ligand binding. MET-106, LEU-356 and TYR-103 residues exhibit the most favorable energetic profiles. This finding aligns with the visual inspection results, corroborating the significance of hydrophobic interactions in stabilizing the complex. These residues form van der Waals contacts interactions with the ligand, creating a stable interaction with the binding pocket. However, GLU-205 and ARG-361 demonstrate the most destabilizing effects on the complex, primarily due to electrostatic repulsion and unfavorable desolvation. This suggests that these residues may be positioned in a way that creates electrostatic clashes with the ligand or requires energetically costly displacement of water molecules upon binding. The overall analysis confirms the predominance of nonpolar interactions in stabilizing the binding pocket, with specific residues acting as key anchoring points. These findings are valuable for drug design efforts that utilize natural triterpene structures, like those examined in this study, as it highlights potential targets for optimization to improve binding affinity and specificity.

**Figure 7:**
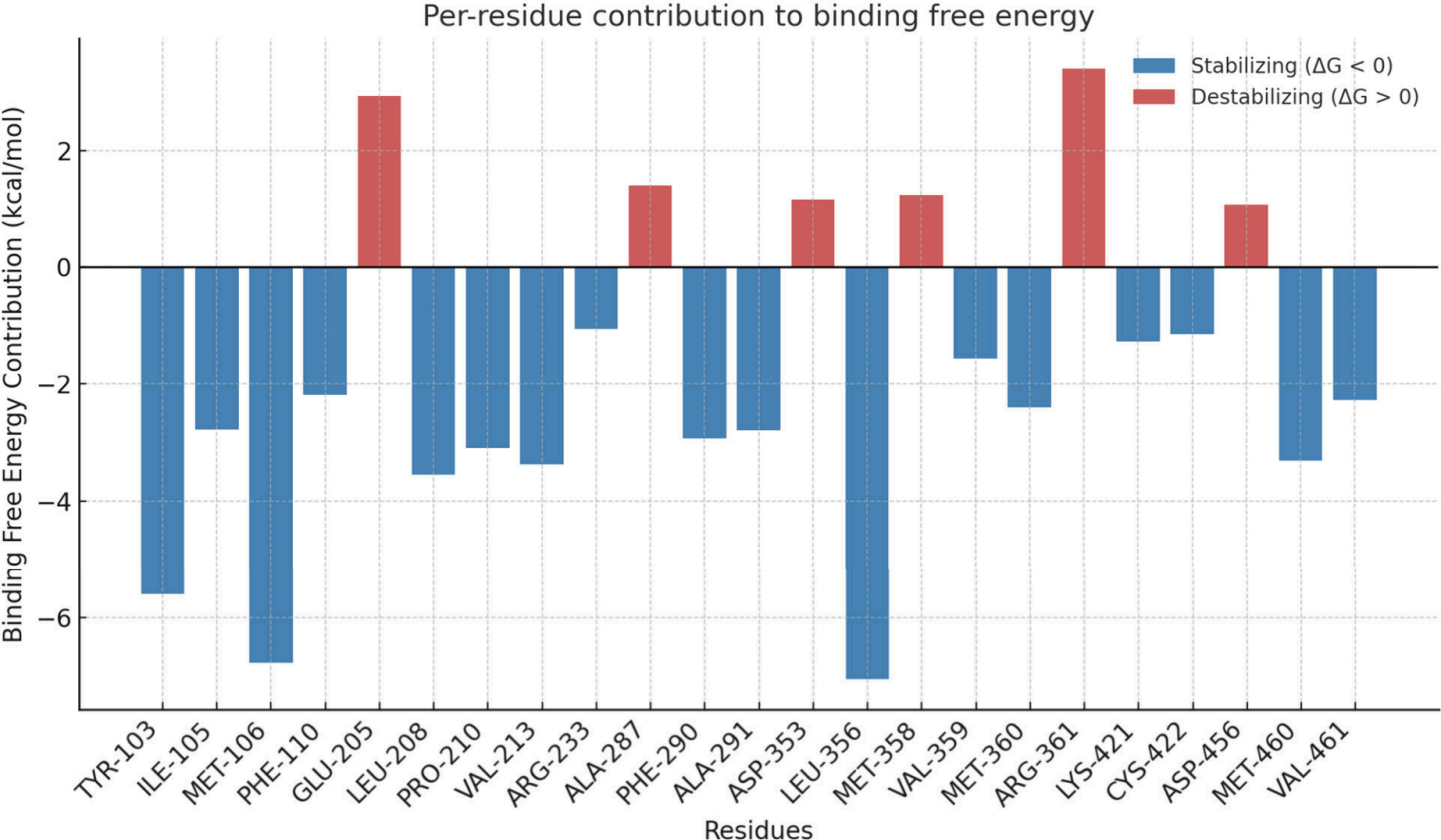
Binding free energy analysis of the ligand–protein complex. (A) Per-residue decomposition of binding free energy contributions, highlighting stabilizing (negative) and destabilizing (positive) residues.

## 4 Final Considerations

In response to a dire need for new drugs to treat Chagas disease a subfraction isolated from aerial parts of *M. campanulata* rich in aliphatic hydrocarbons and pentacyclic triterpenes was screened against the epimastigote forms of *T. cruzi*. Since the subfraction was active against epimastigote forms it can be tested on forms developed in vertebrate hosts (amastigotes and trypomastigotes). In summary, the study demonstrated that pseudotaraxasterol is a strong candidate for *T. cruzi* inhibition, binding stably and with high affinity to the CYP51 (4C27) target, with interactions dominated by hydrophobic forces under simulated molecular dynamics conditions. Our results contribute to a better understanding of the Brazil’s plant biodiversity, as many species are still poorly studied in terms of their chemical composition and biological potential. The present study indicates that these natural sources may become important sources for therapeutic agents.

## Supplementary Material



## Notes

NMR and GC–MS spectra, Ramachandran plots, DFT optimized geometries (PDF)
