# Supplementary material for: In Vitro and In Silico Evaluation of the Trypanocidal Activity of a Subfraction Isolated from Mutisia campanulata
Source: ACS Omega. 2026 Jan 23;11(5):7659–71. doi: 10.1021/acsomega.5c08989 (PMC12903033; doi:10.1021/acsomega.5c08989)
Supplement: Supplementary file 1 [file ao5c08989_si_001.pdf]

In Vitro and *In Silico* Evaluation of the Trypanocidal Activity of a Subfraction Isolated from *Mutisia campanulata*

Grazielle Pereira da Silva[a], Lucas Resende Dutra Sousa[b], Paula Melo de Abreu Vieira[c], Ricardo Stefani[a], and Andréa Mendes do Nascimento[a,]*

[a] *Department of Chemistry, Federal University of Ouro Preto (UFOP), Ouro Preto, MG, Brazil*

[b] *Phytotechnology Laboratory, School of Pharmacy, Federal University of Ouro Preto (UFOP), Ouro Preto, MG, Brazil*

[c] *Morphopathology Laboratory, Department of Biological Sciences, Federal University of Ouro Preto (UFOP), Ouro Preto, MG, Brazil*

*Email: andnascimento@ufop.edu.br

Key words: *Mutisia campanulata*, pentacyclic triterpenes, trypanocidal, epimastigote, pseudotaraxasterol.

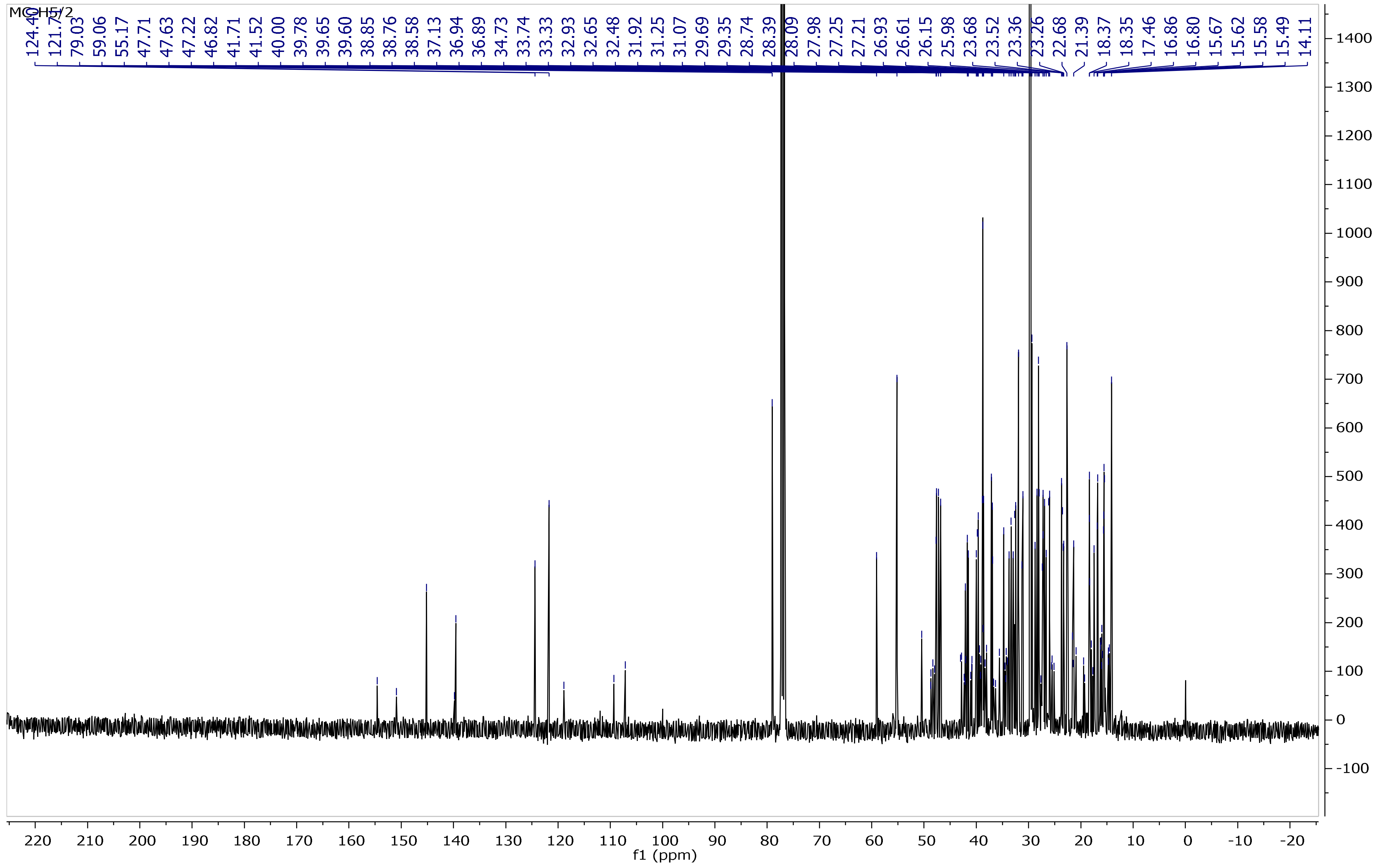

Figure S1: ^{13}C NMR (100 MHz) spectrum of the subfraction MCFr5 in $CDCl_3$

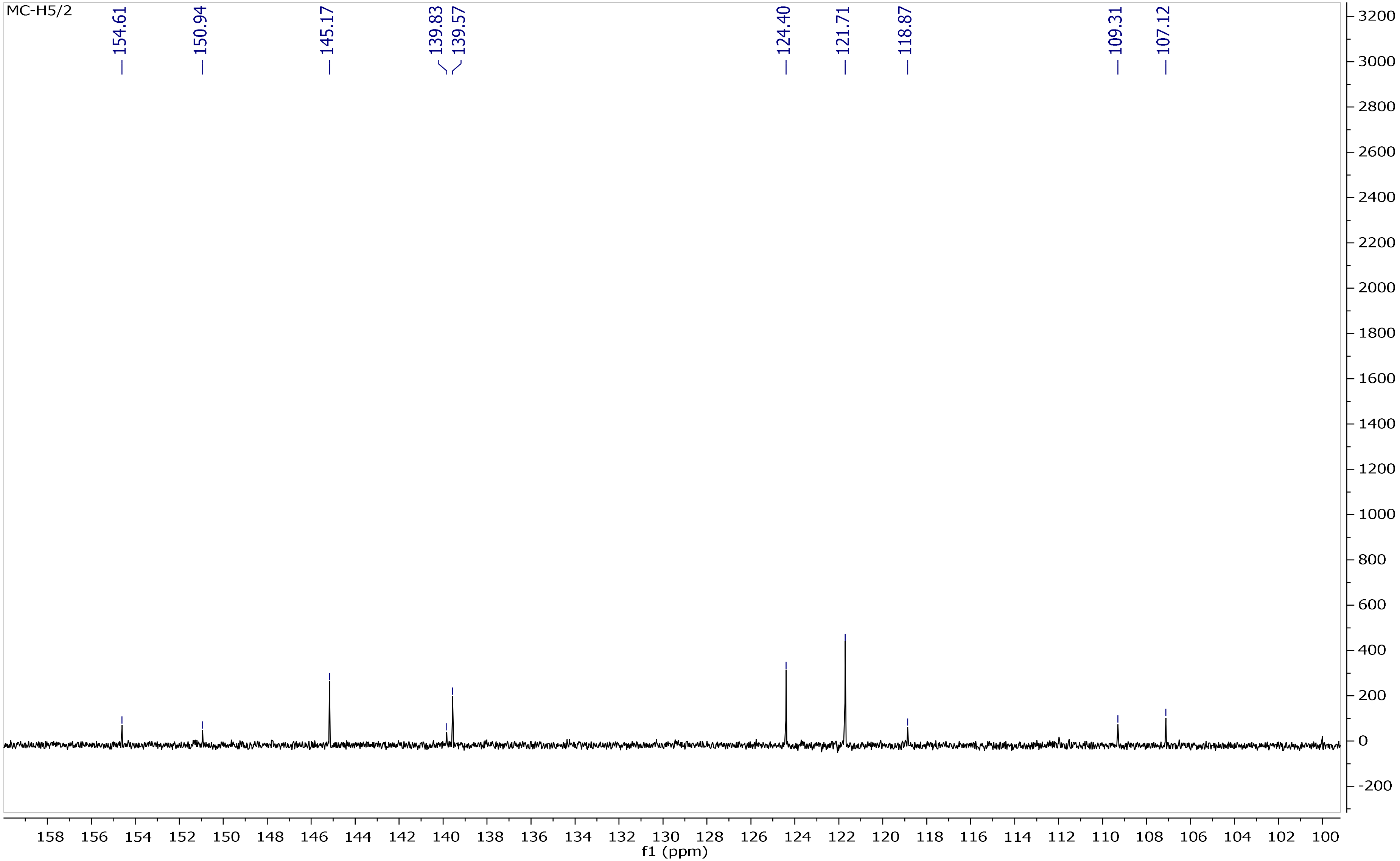

Figure S2: ^{13}C NMR (100 MHz) spectrum of the subfraction MCFr5 in $CDCl_3$ (expansion)

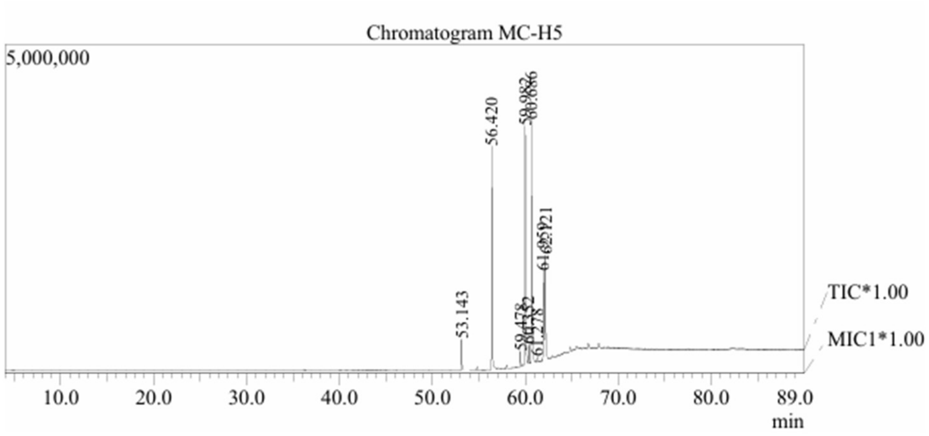

Peak Report TIC

Peak#	R.Time	Area	Area%	Name
1	53.143	1608939	2.22	
2	56.420	12701283	17.54	
3	59.478	751732	1.04	
4	59.982	19857427	27.43	Beta.-Amyrin
5	60.352	945862	1.31	4,22-Cholestadien-3-one
6	60.686	19723248	27.24	Alfa-amirina
7	61.278	224691	0.31	Stigmast-4-en-3-one
8	61.959	6957291	9.61	Pseudotaraxasterol
9	62.121	9632862	13.30	Taraxasterol

Figure S3: GC-MS of the subfraction MCFr5

Library

<< Target >>
Line#:1 R.Time:53.145(Scan#:9830) MassPeaks:56
RawMode:Averaged 53.140-53.150(9829-9831) BasePeak:57.10(75999)
BG Mode:Calc. from Peak

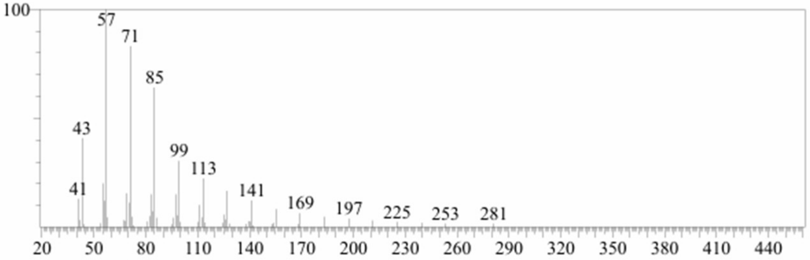

Hit#:1 Entry:304717 Library:WILEY7.LIB
SI:95 Formula:C32 H66 CAS:544-85-4 MolWeight:451 RetIndex:0
CompName:Dotriacontane (CAS) n-Dotriacontane $$ Bicetyl $$ Tris(trimethylsilyl)ether, methyl ester of etl

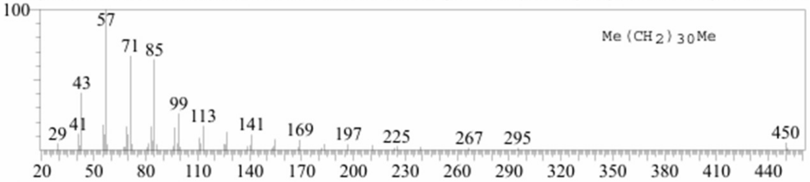

Hit#:2 Entry:30293 Library:NIST11s.lib
SI:95 Formula:C32H66 CAS:544-85-4 MolWeight:450 RetIndex:3202
CompName:Dotriacontane $$ n-Dotriacontane $$ Bicetyl $$

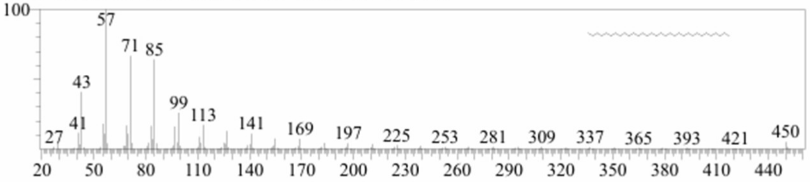

Hit#:3 Entry:1423 Library:FFNSC1.3.lib
SI:95 Formula:C30 H62 CAS:638-68-6 MolWeight:422 RetIndex:3000
CompName:Triacontane <n->

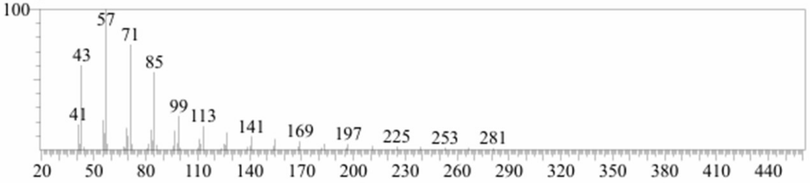

<< Target >>
Line#:2 R.Time:56.420(Scan#:10485) MassPeaks:113
RawMode:Averaged 56.415-56.425(10484-10486) BasePeak:57.10(506220)
BG Mode:Calc. from Peak

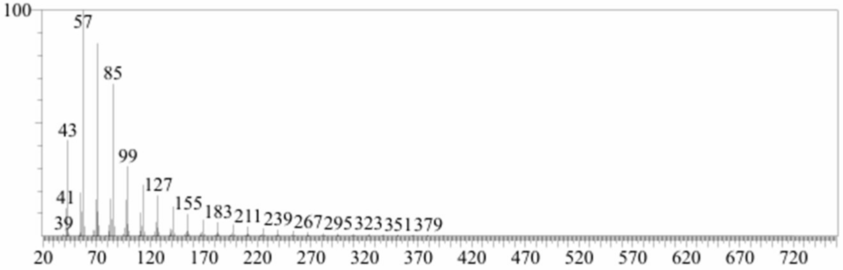

Hit#:1 Entry:30293 Library:NIST11s.lib
SI:96 Formula:C32H66 CAS:544-85-4 MolWeight:450 RetIndex:3202
CompName:Dotriacontane $$ n-Dotriacontane $$ Bicetyl $$

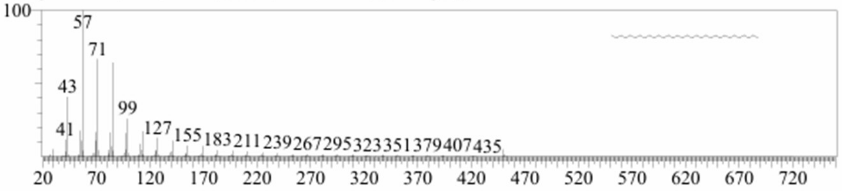

Hit#:2 Entry:304717 Library:WILEY7.LIB
SI:95 Formula:C32 H66 CAS:544-85-4 MolWeight:451 RetIndex:0
CompName:Dotriacontane (CAS) n-Dotriacontane $$ Bicetyl $$ Tris(trimethylsilyl)ether, methyl ester of etl

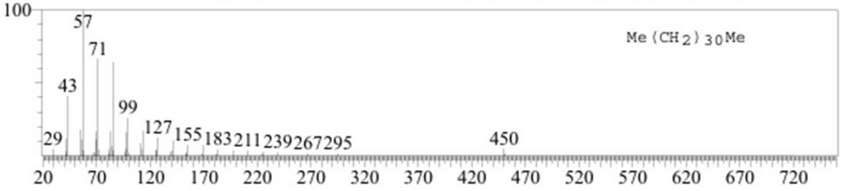

Hit#:3 Entry:30904 Library:NIST11s.lib
SI:95 Formula:C54H110 CAS:5856-66-6 MolWeight:758 RetIndex:5389
CompName:Tetrapentacontane

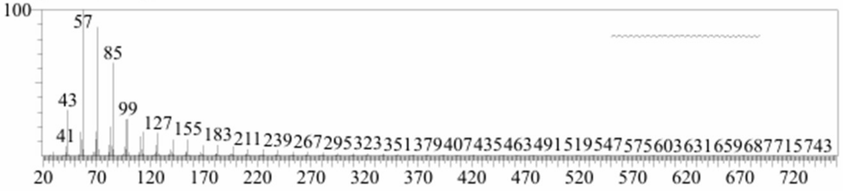

<< Target >>
Line#:3 R.Time:59.480(Scan#:11097) MassPeaks:67
RawMode:Averaged 59.475-59.485(11096-11098) BasePeak:57.10(29589)
BG Mode:Calc. from Peak

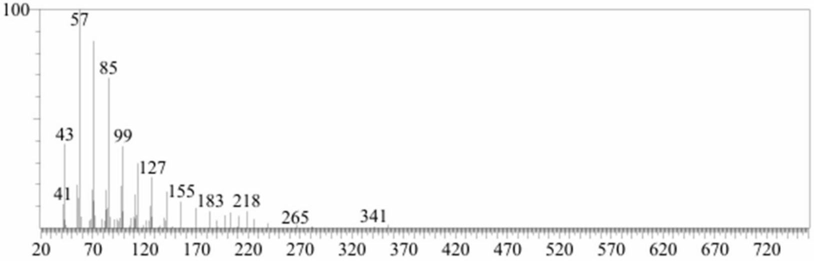

Hit#:1 Entry:30904 Library:NIST11s.lib
SI:90 Formula:C54H110 CAS:5856-66-6 MolWeight:758 RetIndex:5389
CompName:Tetrapentacontane

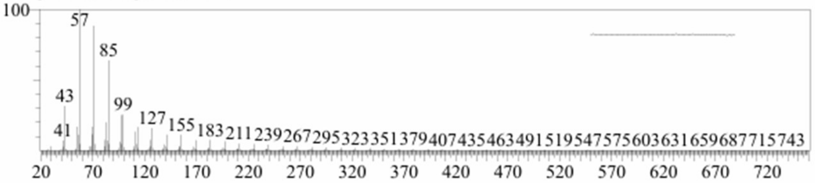

Hit#:2 Entry:335793 Library:WILEY7.LIB
SI:90 Formula:C54 H110 CAS:5856-66-6 MolWeight:759 RetIndex:0
CompName:TETRAPENTACOSAN $$ TETRAPENTACONTANE $$

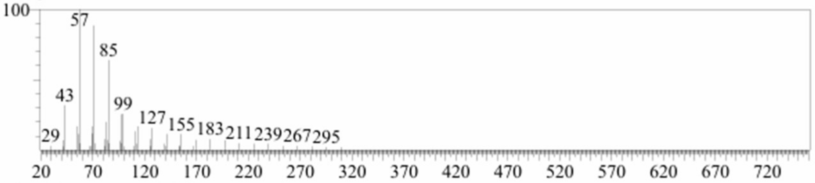

Hit#:3 Entry:304717 Library:WILEY7.LIB
SI:89 Formula:C32 H66 CAS:544-85-4 MolWeight:451 RetIndex:0
CompName:Dotriacontane (CAS) n-Dotriacontane $$ Bicetyl $$ Tris(trimethylsilyl)ether, methyl ester of etl

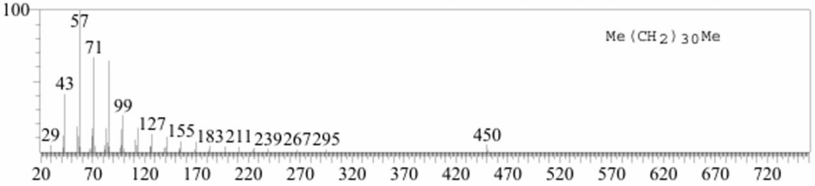

<< Target >>
Line#:4 R.Time:59.980(Scan#:11197) MassPeaks:185
RawMode:Averaged 59.975-59.985(11196-11198) BasePeak:218.25(512273)
BG Mode:Calc. from Peak

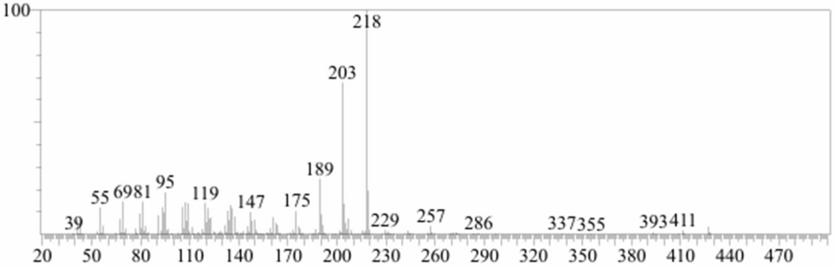

Hit#:1 Entry:191127 Library:NIST11.lib
SI:93 Formula:C30H50O CAS:559-70-6 MolWeight:426 RetIndex:2886
CompName:.beta.-Amyrin $$ Olean-12-en-3-ol, (3.beta.)- $$ Olean-12-en-3.beta.-ol $$ .beta.-Amyrenol $$

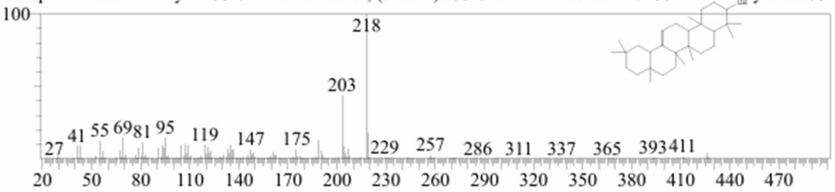

Hit#:2 Entry:58 Library:MY LIBRARY.lib
SI:93 Formula:C30 H50 O CAS:559-70-6 MolWeight:426 RetIndex:0
CompName:Beta-amirina

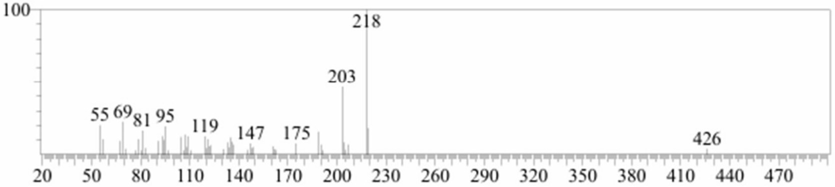

Hit#:3 Entry:32 Library:MY LIBRARY.lib
SI:91 Formula:C30 H50 O CAS:559-70-6 MolWeight:426 RetIndex:0
CompName:Beta-amirina

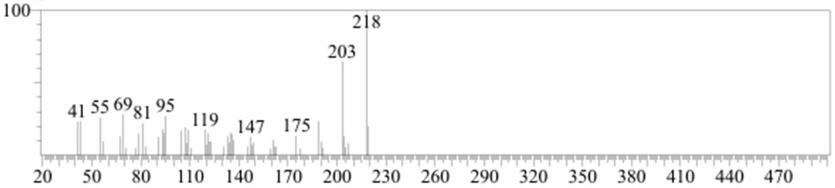

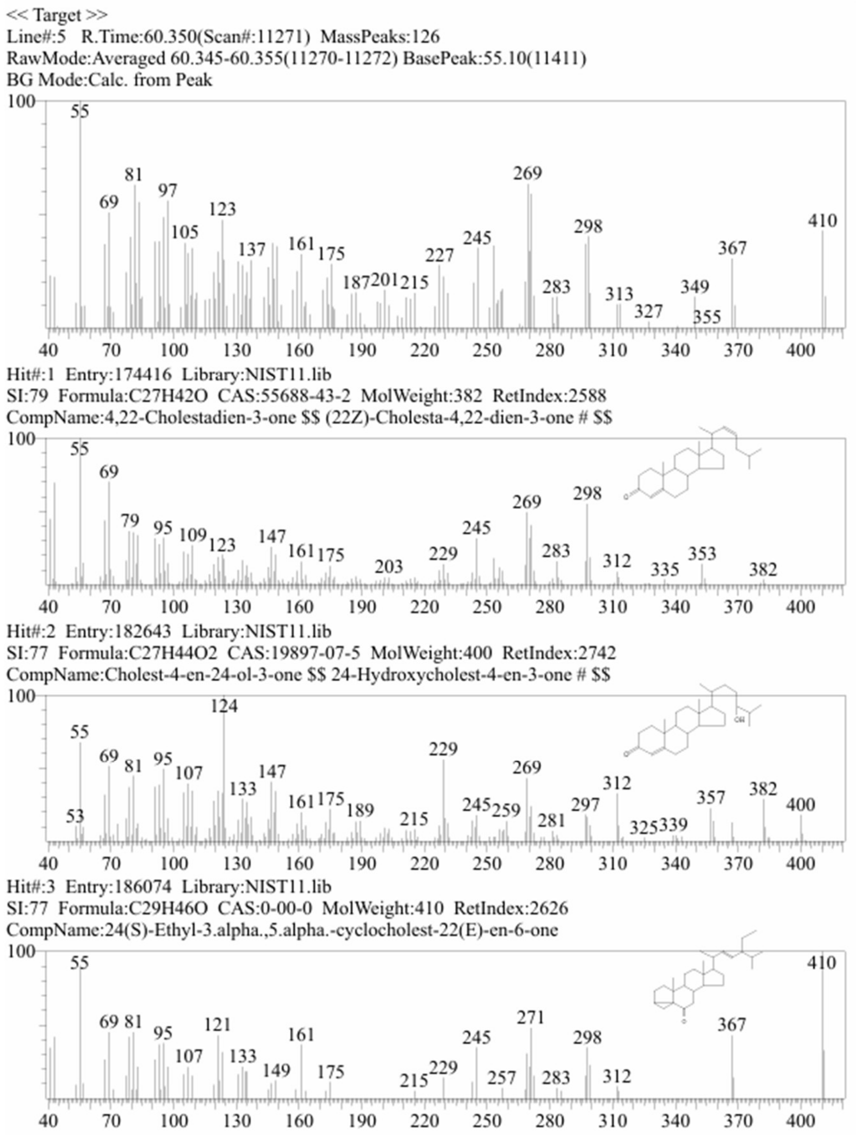
<< Target >>
Line#:5 R.Time:60.350(Scan#:11271) MassPeaks:126
RawMode:Averaged 60.345-60.355(11270-11272) BasePeak:55.10(11411)
BG Mode:Calc. from Peak
Hit#:1 Entry:174416 Library:NIST11.lib
SI:79 Formula:C27H42O CAS:55688-43-2 MolWeight:382 RetIndex:2588
CompName:4,22-Cholestadien-3-one $$ (22Z)-Cholesta-4,22-dien-3-one # $$
Hit#:2 Entry:182643 Library:NIST11.lib
SI:77 Formula:C27H44O2 CAS:19897-07-5 MolWeight:400 RetIndex:2742
CompName:Cholest-4-en-24-ol-3-one $$ 24-Hydroxycholest-4-en-3-one # $$
Hit#:3 Entry:186074 Library:NIST11.lib
SI:77 Formula:C29H46O CAS:0-00-0 MolWeight:410 RetIndex:2626
CompName:24(S)-Ethyl-3.alpha.,5.alpha.-cyclocholest-22(E)-en-6-one

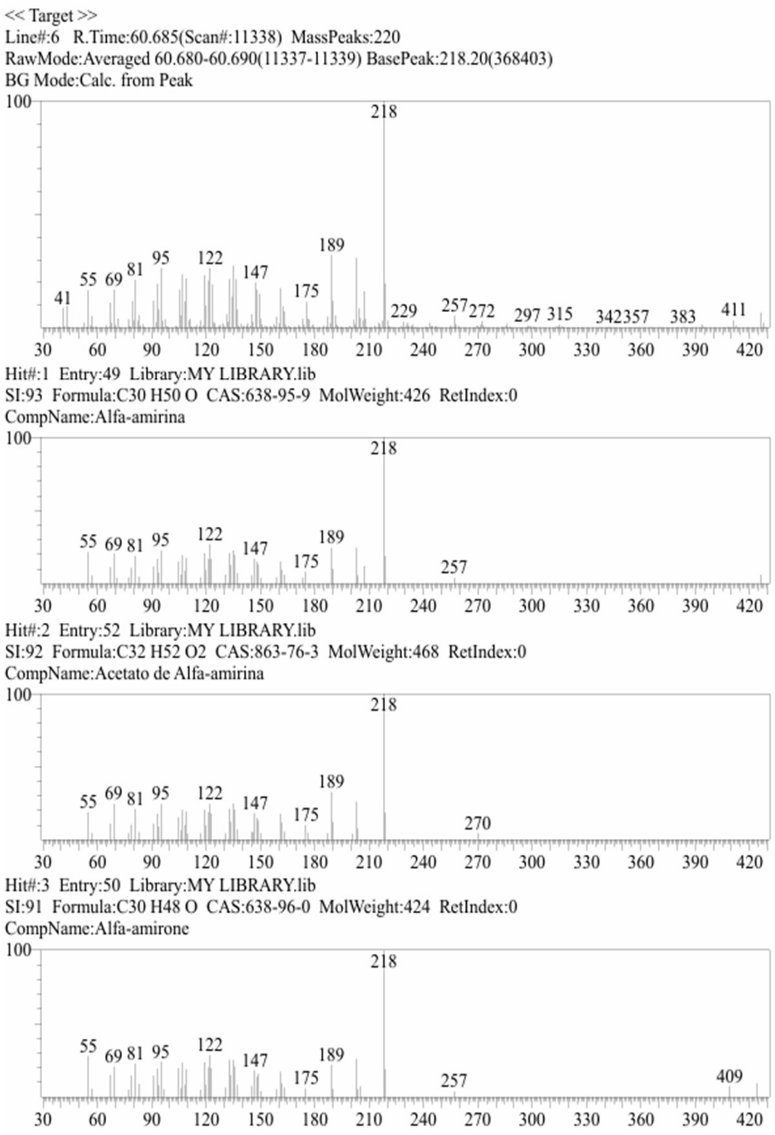
<< Target >>
Line#:6 R.Time:60.685(Scan#:11338) MassPeaks:220
RawMode:Averaged 60.680-60.690(11337-11339) BasePeak:218.20(368403)
BG Mode:Calc. from Peak
Hit#:1 Entry:49 Library:MY LIBRARY.lib
SI:93 Formula:C30 H50 O CAS:638-95-9 MolWeight:426 RetIndex:0
CompName:Alfa-amirina
Hit#:2 Entry:52 Library:MY LIBRARY.lib
SI:92 Formula:C32 H52 O2 CAS:863-76-3 MolWeight:468 RetIndex:0
CompName:Acetato de Alfa-amirina
Hit#:3 Entry:50 Library:MY LIBRARY.lib
SI:91 Formula:C30 H48 O CAS:638-96-0 MolWeight:424 RetIndex:0
CompName:Alfa-amirone

<< Target >>
Line#:7 R.Time:61.275(Scan#:11456) MassPeaks:74
RawMode:Averaged 61.270-61.280(11455-11457) BasePeak:124.15(6862)
BG Mode:Calc. from Peak

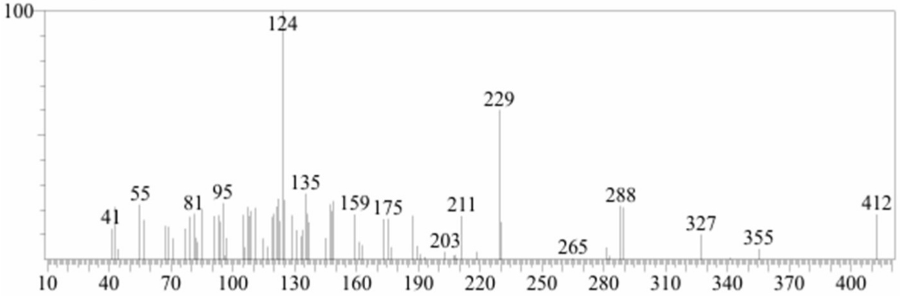

Hit#:1 Entry:186827 Library:NIST11.lib
SI:79 Formula:C29H48O CAS:1058-61-3 MolWeight:412 RetIndex:2714
CompName:Stigmast-4-en-3-one $$ 4-Stigmasten-3-one $$ Sitostenone $$ .DELTA.4-Sitosterol-3-one $$

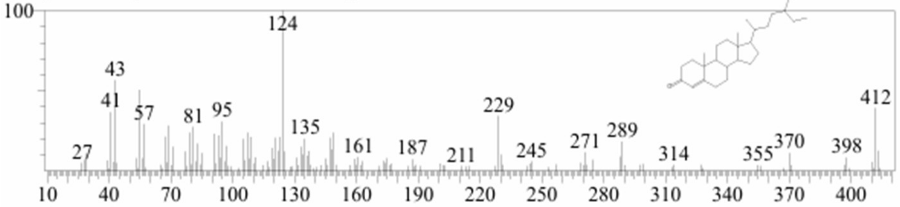

Hit#:2 Entry:175451 Library:NIST11.lib
SI:79 Formula:C27H44O CAS:601-57-0 MolWeight:384 RetIndex:2580
CompName:Cholest-4-en-3-one $$ 3-Oxocholest-4-ene $$ Cholestenone $$ .delta.4-Cholesten-3-one $$ .del

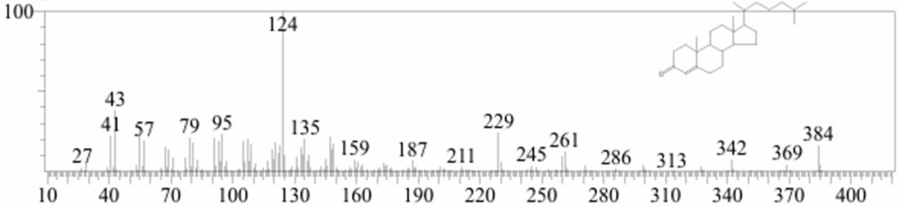

Hit#:3 Entry:290260 Library:WILEY7.LIB
SI:79 Formula:C29 H48 O CAS:1058-61-3 MolWeight:412 RetIndex:0
CompName:Stigmast-4-en-3-one (CAS) 4-Stigmasten-3-one $$ .DELTA.4-Sitosterol-3-one $$ (24R)-4-STI(

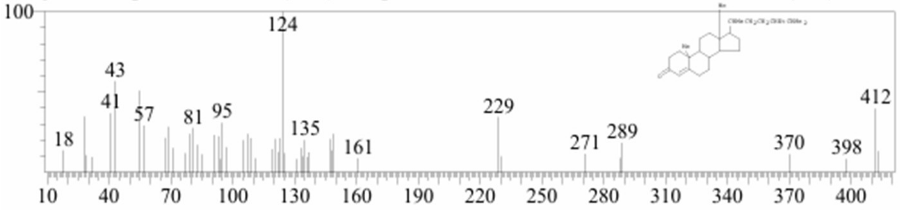

<< Target >>
Line#:8 R.Time:61.960(Scan#:11593) MassPeaks:167
RawMode:Averaged 61.955-61.965(11592-11594) BasePeak:189.20(88816)
BG Mode:Calc. from Peak

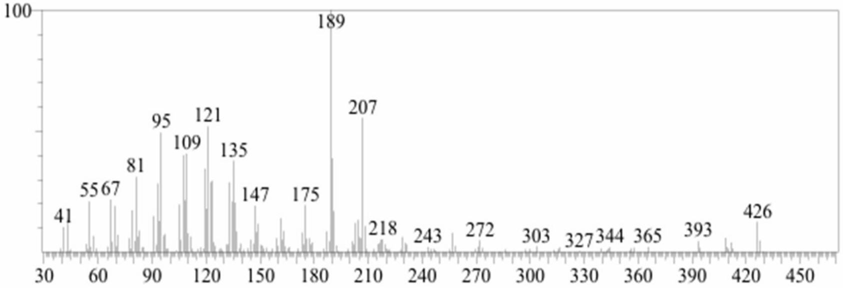

Hit#:1 Entry:19 Library:MY LIBRARY.lib
SI:90 Formula: CAS:0-00-0 MolWeight:426 RetIndex:0
CompName:pseudotaraxasterol

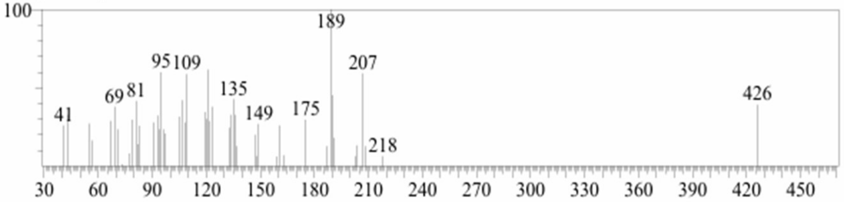

Hit#:2 Entry:14 Library:MY LIBRARY.lib
SI:88 Formula:C32 H52 O2 CAS:4586-65-6 MolWeight:468 RetIndex:0
CompName:Acetato pseudotaraxasterol

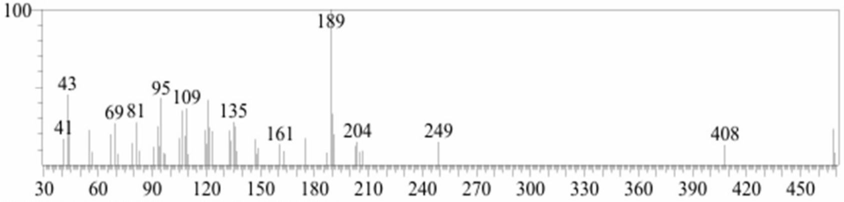

Hit#:3 Entry:15 Library:MY LIBRARY.lib
SI:85 Formula:C30 H52 O2 CAS:6426-43-3 MolWeight:468 RetIndex:0
CompName:Acetato taraxasterol

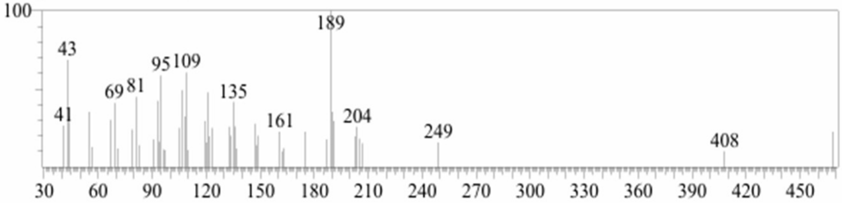

<< Target >>
Line#:9 R.Time:62.120(Scan#:11625) MassPeaks:180
RawMode:Averaged 62.115-62.125(11624-11626) BasePeak:189.20(67224)
BG Mode:Calc. from Peak

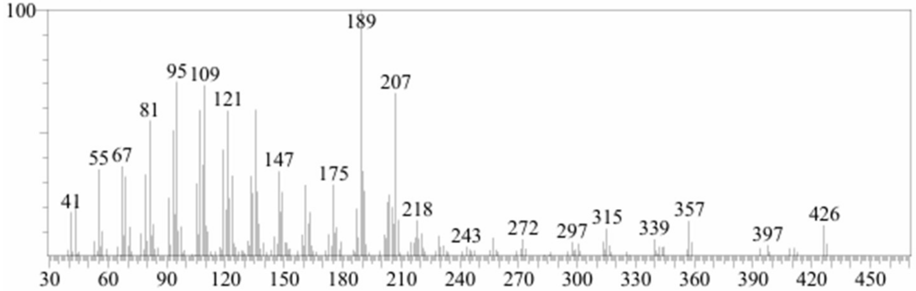

Hit#:1 Entry:19 Library:MY LIBRARY.lib
SI:89 Formula: CAS:0-00-0 MolWeight:426 RetIndex:0
CompName:pseudotaraxasterol

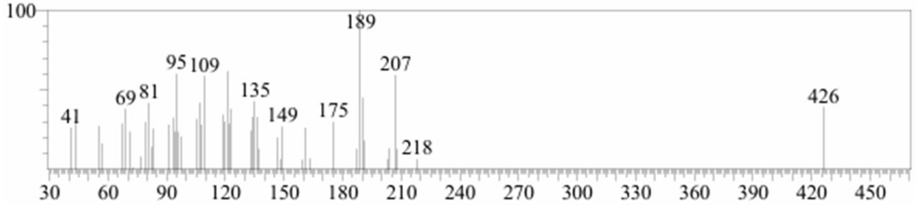

Hit#:2 Entry:53 Library:MY LIBRARY.lib
SI:88 Formula:C30 H50 O CAS:545-47-1 MolWeight:426 RetIndex:0
CompName:Lupeol

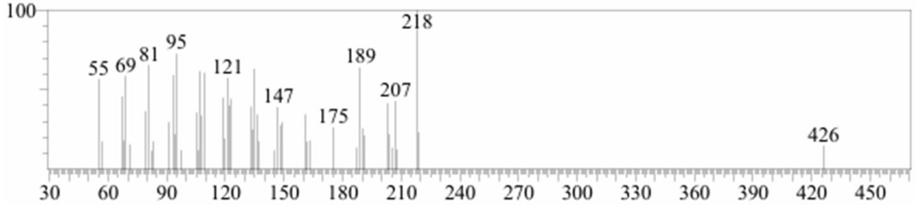

Hit#:3 Entry:15 Library:MY LIBRARY.lib
SI:88 Formula:C30 H52 O2 CAS:6426-43-3 MolWeight:468 RetIndex:0
CompName:Acetato taraxasterol

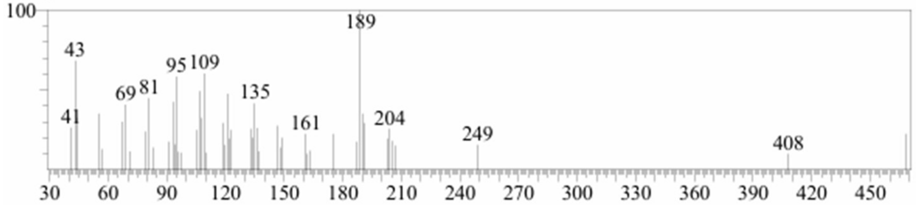

Sample Information

Analyzed by	: Admin
Analyzed	: 24/07/2019 20:40:28
Sample Type	: Unknown
Level #	: 1
$EndIf$IS Amount	: [1]=1
Sample Amount	: 1
Dilution Factor	: 1
Vial #	: 4
Injection Volume	: 1.00
Data File	: C:\GCMSsolution\Data\Project1\Andréa\Pad Hidrocarb-24-07.qgd
Method File	: C:\GCMSsolution\Data\Project1\Andréa\Fiehn mod_Pad.qgm
Org Method File	: C:\GCMSsolution\Data\Project1\Andréa\Fiehn mod_Pad.qgm
Report File	:
Tuning File	: C:\GCMSsolution\System\Tune1\2019\05-07-2019.qgt

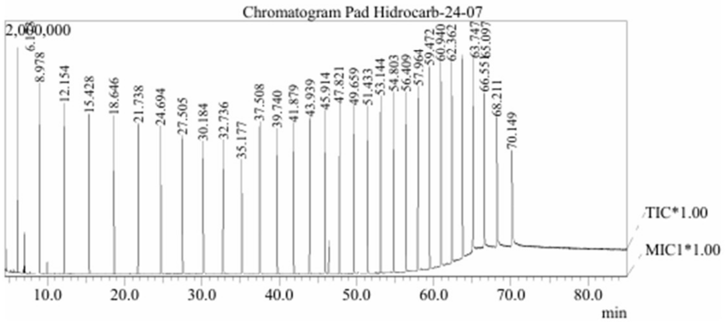

Peak Report TIC

Peak#	R.Time	Area	Area%	Name
1	6.173	3387097	2.65	Decane <n->
2	8.978	3343571	2.61	Undecane
3	12.154	3289519	2.57	Dodecane
4	15.428	3335227	2.61	Tridecane <n->
5	18.646	3205669	2.50	Tetradecane (CAS) n-Tetradecane
6	21.738	3310645	2.59	Pentadecane (CAS) n-Pentadecane
7	24.694	3230535	2.52	Hexadecane <n->
8	27.505	3026114	2.36	Heptadecane <n->
9	30.184	3089566	2.41	Octadecane <n->
10	32.736	3188236	2.49	Nonadecane <n->
11	35.177	2730387	2.13	Eicosane <n->
12	37.508	3602962	2.81	Heneicosane <n->
13	39.740	3629790	2.84	Docosane <n->
14	41.879	3804210	2.97	Tricosane <n->
15	43.939	3949787	3.09	Tetracosane <n->
16	45.914	3946486	3.08	Pentacosane <n->
17	47.821	4227660	3.30	Hexacosane <n->
18	49.659	4249577	3.32	Heptacosane <n->
19	51.433	4346958	3.40	Octacosane
20	53.144	4618030	3.61	Nonacosane
21	54.803	4800231	3.75	TriacontaneUndecaneDodecaneTridecan
22	56.409	4737630	3.70	HentriacontaneUndecaneDodecaneTride
23	57.964	5105113	3.99	Dotriacontane
24	59.472	5286609	4.13	Tritriacontane
25	60.940	5483899	4.28	Tetratriacontane
26	62.362	5251708	4.10	Pentatriacontane
27	63.747	5708237	4.46	Hexatriacontane
28	65.097	5345064	4.18	Heptatriacontane
29	66.551	5510521	4.30	Octatriacontane
30	68.211	4888718	3.82	Nonatriacontane
31	70.149	4388863	3.43	Tetracontane
		128018619	100.00	

Figure S4: GC-MS of a series of *n*-alkanes

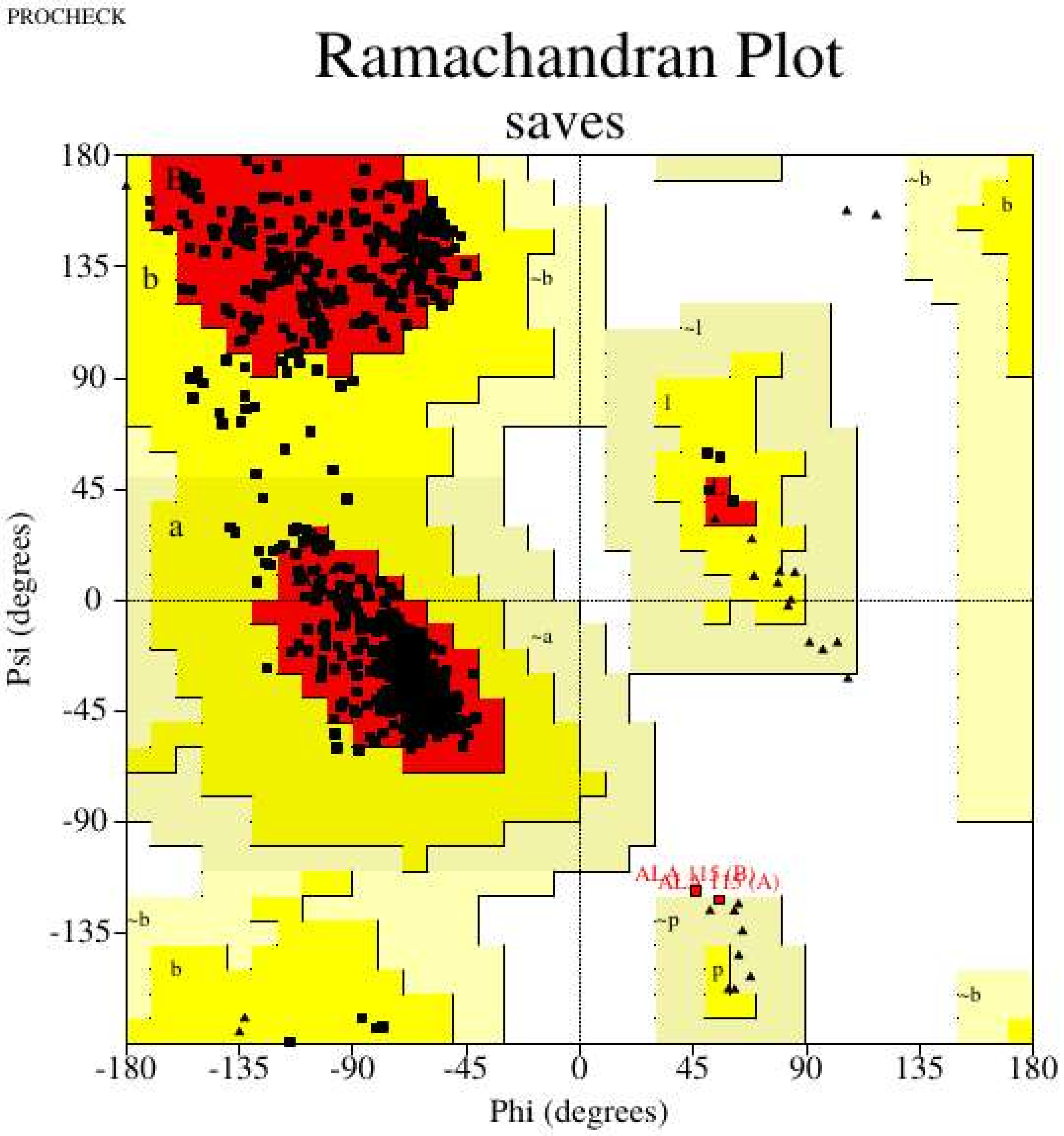

Figure S5: Ramachandran plot of the initial 4C27 protein structure, generated by PROCHECK

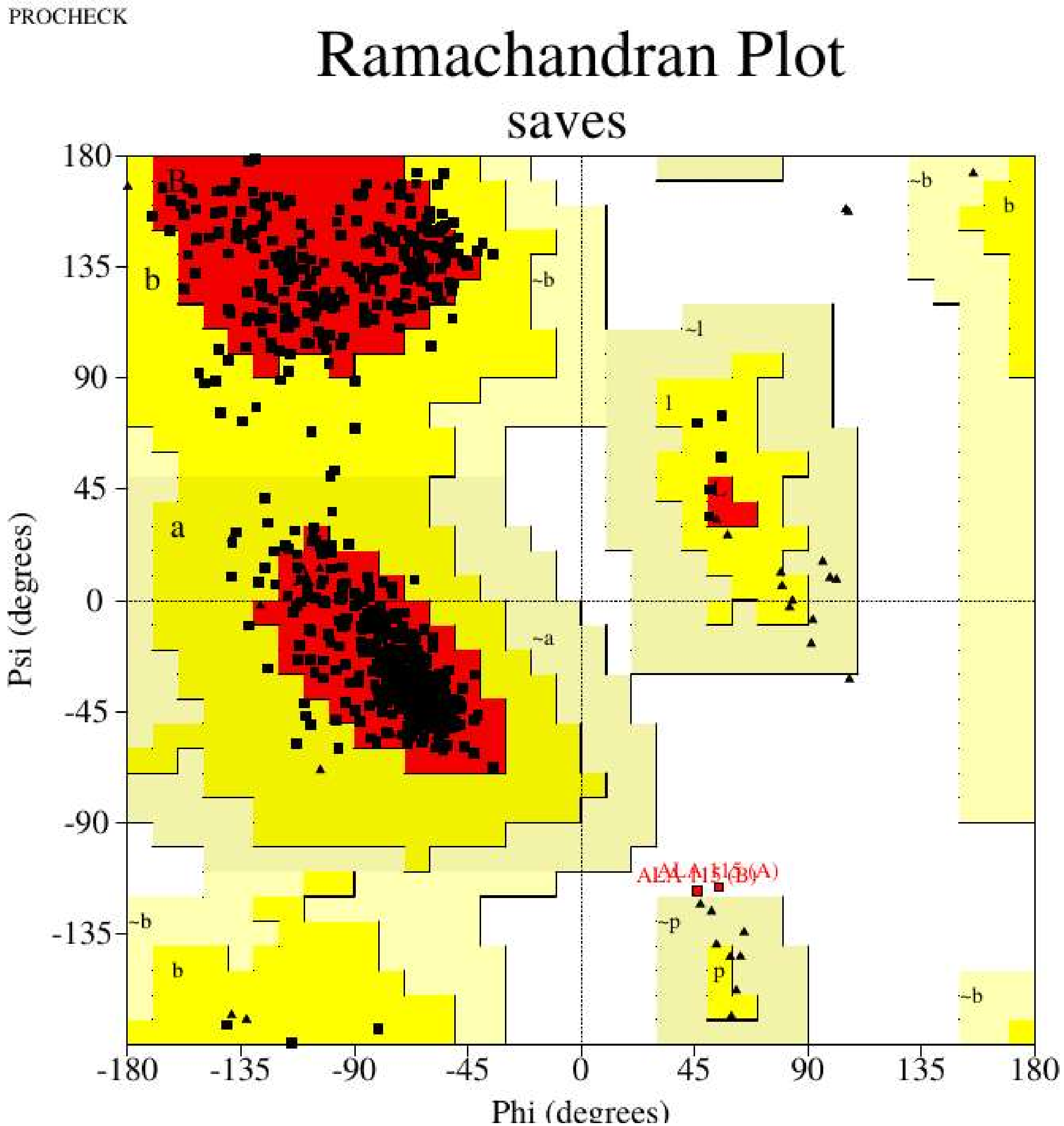

Figure S6: Ramachandran plot of the final 4C27 protein structure, generated by PROCHECK

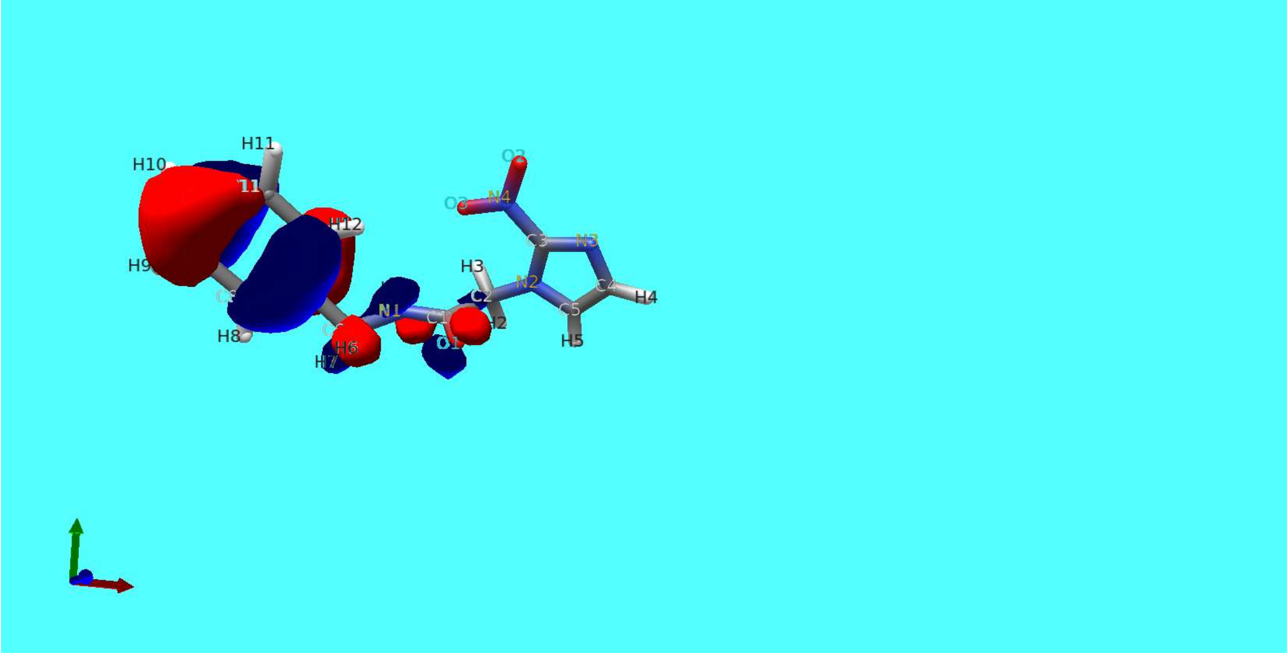

Figure S7: DFT-optimized geometry of the control compound, 2-Benzinadole, displaying the isodensity surface of the Highest Occupied Molecular Orbital (HOMO)

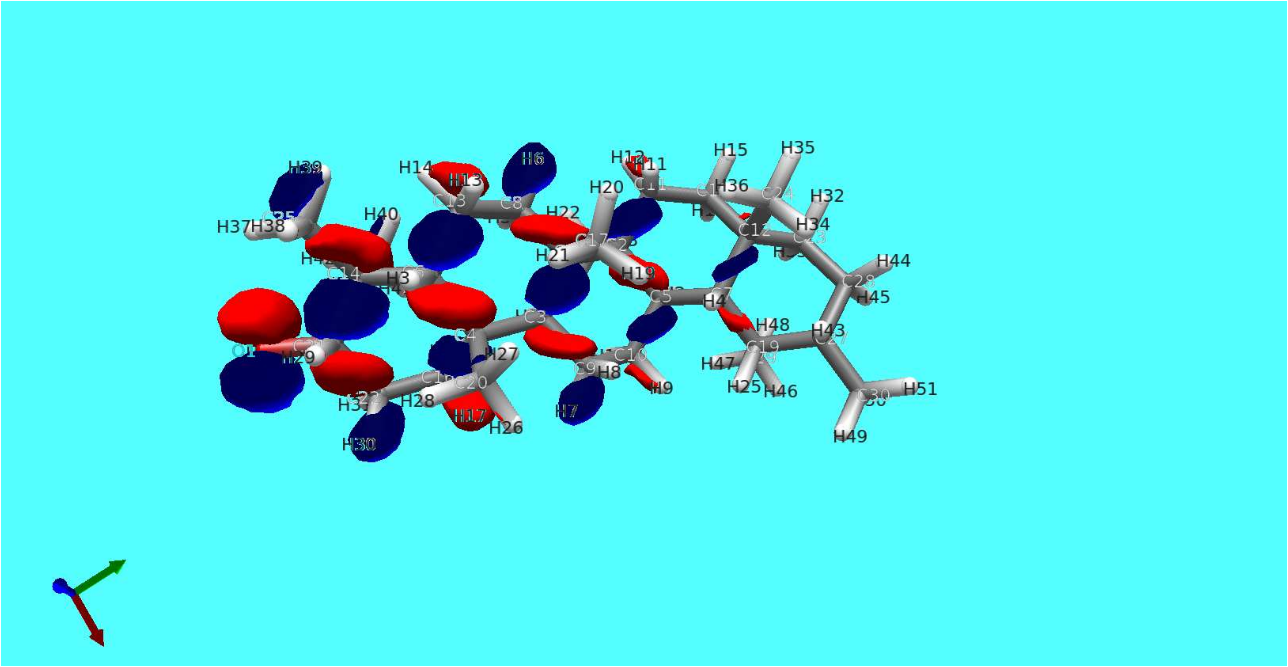

Figure S8: DFT-optimized geometry of pseudotaraxasterol, displaying the isodensity surface of the Highest Occupied Molecular Orbital (HOMO)
